# G3BP1 inhibits ubiquitinated protein aggregations induced by p62 and USP10

**DOI:** 10.1038/s41598-019-46237-1

**Published:** 2019-09-09

**Authors:** Sergei Anisimov, Masahiko Takahashi, Taichi Kakihana, Yoshinori Katsuragi, Hiroki Kitaura, Lu Zhang, Akiyoshi Kakita, Masahiro Fujii

**Affiliations:** 1 Division of Virology, Niigata University Graduate School of Medical and Dental Sciences, Niigata, 951-8510 Japan; 2 Department of Pathology, Brain Research Institute, University of Niigata, Niigata, 951-8585 Japan

**Keywords:** Mechanisms of disease, Parkinson's disease

## Abstract

The aberrant accumulation of ubiquitinated protein aggregates in cells plays a critical role in the pathogenesis of several degenerative diseases, including Parkinson disease (PD) and cystic fibrosis (CF). In this study, we found that Ras GTPase-activating protein-binding protein 1 (G3BP1) inhibits ubiquitinated protein aggregations induced by p62 and USP10 in cultured cells. p62 is a ubiquitin receptor, and p62 and its binding partner USP10 have been shown to augment ubiquitinated protein aggregation. G3BP1 interacted with p62 and USP10 and inhibited p62/USP10-induced protein aggregation. The G3BP1 inhibition of protein aggregations targeted two aggregation-prone proteins, α-synuclein and CFTR-ΔF508, which are causative factors of PD and CF, respectively. G3BP1 depletion increased the amounts of ubiquitinated α-synuclein and CFTR-ΔF508 protein. A proteasome reporter indicated that G3BP1 depletion inhibits the proteasome activity. We herein present evidence that G3BP1, p62 and USP10 together control ubiquitinated protein toxicity by controlling both ubiquitination and aggregation. Taken together, these results suggest that G3BP1, p62 and USP10 could be therapeutic targets for ubiquitinated protein aggregation disorders, including PD and CF.

## Introduction

The aberrant accumulation of ubiquitinated protein aggregates in cells is a common cause of many degenerative diseases, such as α-synuclein in Parkinson’s disease (PD) and CFTR-ΔF508 in cystic fibrosis (CF)^1–3^. α-synuclein is a causative factor of familial and sporadic PD^4^. α-synuclein is aggregation-prone, and α-synuclein-associated PD is characterized by intracellular deposition of ubiquitinated α-synuclein aggregates as a Lewy body in neurons in brain lesions^4,5^. CF is a genetic disease caused by mutations in the cystic fibrosis transmembrane conductance regulator (CFTR) gene^6^. CFTR-ΔF508 is the most frequent CFTR-causing mutant, with one amino acid deletion in CFTR^7^. CFTR-ΔF508 is also ubiquitination-prone, and ubiquitinated CFTR-ΔF508 is degraded by the ubiquitin-proteasome system and reduces the amount of functional CFTR^8^. However, precisely how such cells control the ubiquitinated protein aggregations and how such pathogenic inclusions are formed in degenerative diseases remains poorly understood.

Many ubiquitination/aggregation-prone proteins, including α-synuclein and CFTR-ΔF508, share one notable characteristic: they are localized in aggresomes in cultured cells^9,10^. Aggresomes are large cytoplasmic protein aggregates induced by various stress inducers, such as proteasome inhibitor^8,9,11^. Aggresomes contain both ubiquitinated and non-ubiquitinated proteins, autophagy-associated proteins, chaperons and others. They are colocalized with lysosomes^12^, and many aggresome-localizing proteins are degraded by the lysosome-autophagy system. Aggresome formation inhibits apoptosis induced by ubiquitinated proteins^13,14^. Taken together, these results suggest that aggresomes play a protective role against ubiquitinated protein-induced damage to cells.

Ubiquitin-specific protease 10 (USP10) and p62 are key regulators of ubiquitinated protein aggregation and aggresome formation^14^. p62 is a ubiquitin receptor that interacts with ubiquitinated proteins and augments protein aggregation^15^. USP10 has two functions: to promote protein aggregation and aggresome formation^14^. It interacts with p62 and augments p62-induced protein aggregation. In addition, USP10 stimulates the transport of multiple p62-positive ubiquitinated protein aggregates to the perinuclear region to form one big aggregate (aggresome). USP10 is a deubiquitinase for several substrates^16–18^, but the deubiquitinase activity is dispensable for the protein aggregation- and aggresome-inducing activities^14^.

USP10 interacts with two structurally and functionally related proteins: Ras GTPase-activating protein-binding protein 1 (G3BP1) and G3BP2^19,20^. G3BP1 and G3BP2 are RNA-binding proteins and regulate stress granule (SG) formation, a stress-inducible RNA granule^20,21^. Knockdown of G3BP1 and G3BP2 in cultured cells prominently reduces the SG formation induced by various stress stimuli, including arsenite treatment, and the SG-inducing activity of G3BP1 is higher than that of G3BP2 in many cases^20,22^. Of note, G3BP1 knockout mice develop neurodegeneration accompanied by neuronal cell death^23^, suggesting that G3BP1 plays a critical role in the neuronal cell survival.

In this study, we found that G3BP1 is an inhibitor for protein ubiquitination and aggregation in the steady state condition of cells by reducing the ubiquitinated protein aggregation induced by p62 and USP10. This G3BP1 inhibition of ubiquitinated protein aggregations targeted many ubiquitinated proteins including two pathogenic proteins: α-synuclein and CFTR-ΔF508. Furthermore, we found that G3BP1, p62 and USP10 control ubiquitinated protein toxicity by regulating ubiquitination and aggregation. Therefore, our results indicate that G3BP1, p62 and USP10 could be a therapeutic target for ubiquitination-associated diseases including PD and CF.

## Results

### G3BP1 depletion increases the amount of ubiquitinated protein

**Figure 1:**
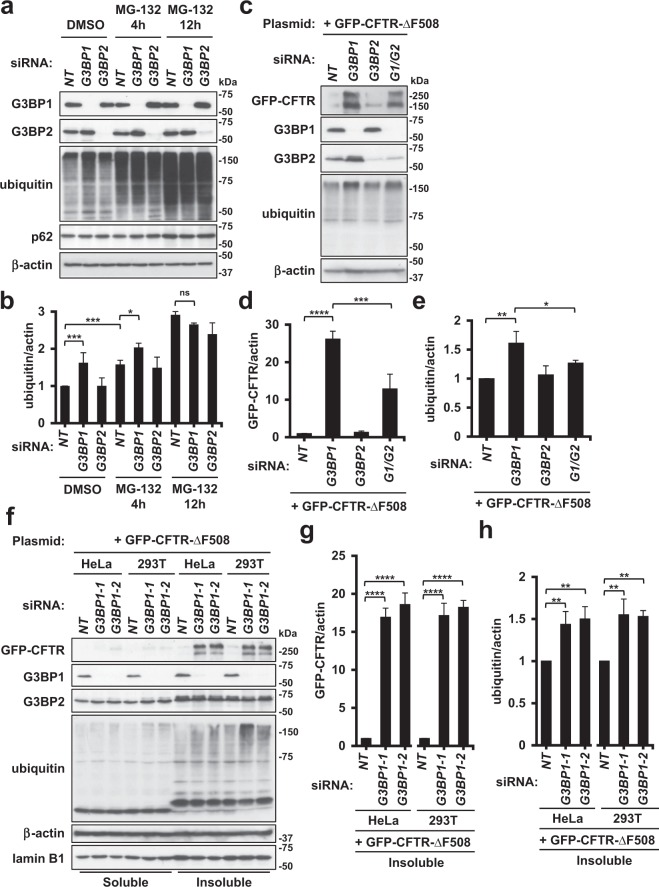
G3BP1 and G3BP2 regulate the amount of ubiquitinated protein. (**a**,**b**) HeLa cells were transfected with G3BP1-siRNA (*G3BP1*), G3BP2-siRNA (*G3BP2*) or the control (*NT*) by lipofection, and the cells were then treated with 5 µM MG-132 for 4-12 h or DMSO. Whole-cell lysates were subjected to a Western blot analysis using anti-G3BP1, anti-G3BP2, anti-ubiquitin, anti-p62 and anti-β-actin antibodies. The ratio of the ubiquitin bands relative to the β-actin one was measured by densitometry scanning, and the means and standard deviation (SD) from three experiments were presented in (**b**). (**c**–**e**) HeLa cells were transfected with G3BP1-siRNA (*G3BP1*), G3BP2-siRNA (*G3BP2*), their combination (*G1*/*G2*) or control (*NT*) by lipofection, and the cells were then transfected with the GFP-CFTR-ΔF508 plasmid. Whole-cell lysates were subjected to a Western blot analysis using anti-GFP (CFTR), anti-G3BP1, anti-G3BP2, anti-ubiquitin and anti-β-actin antibodies. The ratios of the GFP-CFTR band or the ubiquitin one to the β-actin one were presented as the means and SD from three experiments in (**d**,**e**). (**f**–**h**) HeLa and 293T cells were transfected with G3BP1-siRNAs (*G3BP1-1* or *G3BP1-2*) or the control (*NT*) and then transfected with the GFP-CFTR-ΔF508 plasmid. NP-40-soluble and NP-40-insoluble lysates were subjected to a Western blot analysis using anti-GFP (CFTR), anti-G3BP1, anti-G3BP2, anti-ubiquitin, anti-β-actin and anti-lamin B1 antibodies. The ratios of the GFP-CFTR band or ubiquitin one to the β-actin one in the insoluble fractions were presented as the means and SD from three experiments in (**g**,**h**). *P < 0.05; **P < 0.01; ***P < 0.001; ****P < 0.0001; NS: not significant.

USP10 has been shown to augment ubiquitinated protein aggregations by increasing the amount of ubiquitinated proteins^14^. Given that USP10 interacts with G3BP1 and G3BP2^20^, we examined whether or not G3BP1 and G3BP2 regulate ubiquitinated protein aggregation. To this end, we reduced the expressions of G3BP1 and G3BP2 proteins in HeLa cells (G3BP1-knockdown [KD], G3BP2-KD) using small interfering RNA (siRNA) (Fig. 1a,b). A Western blot analysis detected a reduced expression of G3BP1 and G3BP2 proteins in KD cells. These KD cells were treated with the proteasome inhibitor MG-132 for 4 and 12 h. G3BP1-KD increased the amount of ubiquitinated proteins in cells before MG-132 treatment, but the increase was attenuated by MG-132 treatment for 4 and 12 h. In contrast, G3BP2-KD did not show stimulatory activity on the amount of ubiquitinated protein. These results suggested that G3BP1 but not G3BP2 reduces the amount of ubiquitinated proteins in HeLa cells. We noticed that G3BP1-KD increased the amount of G3BP2, whereas G3BP2-KD affected the amount of G3BP1 relatively little. Given that G3BP2 protein is ubiquitination-prone and degraded by proteasome^24^, these results suggested that G3BP1-KD might increase the amount of G3BP2 and ubiquitinated proteins through a similar mechanism.

### G3BP1 depletion augments protein aggregation of CFTR-∆F508

CFTR-ΔF508 is a mutant protein of the cystic fibrosis transmembrane conductance regulator (CFTR) and a causative factor of cystic fibrosis (CF)^6^. CFTR-ΔF508 is a ubiquitination- and aggregation-prone protein that induces dysfunction as a transmembrane conductance regulator, resulting in CF development. Given that CFTR-ΔF508 protein aggregation is augmented by USP10^14^, we examined whether or not G3BP1-KD and/or G3BP2-KD alters the amount and aggregation of CFTR-ΔF508 protein.

GFP-CFTR-ΔF508 is a fusion protein of CFTR-ΔF508 with green fluorescent protein (GFP). GFP-CFTR-ΔF508 plasmid was transfected into G3BP1-KD or G3BP2-KD cells, and the amount of GFP-CFTR-ΔF508 was measured by a Western blot analysis (Fig. 1c–e). G3BP1-KD increased the amount of CFTR-ΔF508, and the increase was attenuated by G3BP2-KD, although G3BP2-KD alone did not markedly affect the amount of CFTR-ΔF508. In addition to CFTR-ΔF508 protein, G3BP1-KD but not G3BP2-KD in the presence of CFTR-ΔF508 increased the amount of ubiquitinated proteins. These results suggested that G3BP1-KD augments the amounts of CFTR-ΔF508 and ubiquitinated proteins by inhibiting the proteasome activity, as examined later. Cell lysate fractionation by a detergent showed that G3BP1-KD increased the amounts of both detergent-soluble and detergent-insoluble CFTR-ΔF508 and ubiquitinated proteins, but the increases in the insoluble fraction were predominant (Fig. 1f–h). Similar results were obtained with two distinct G3BP1-siRNA both in HeLa and 293T cells.

**Figure 2:**
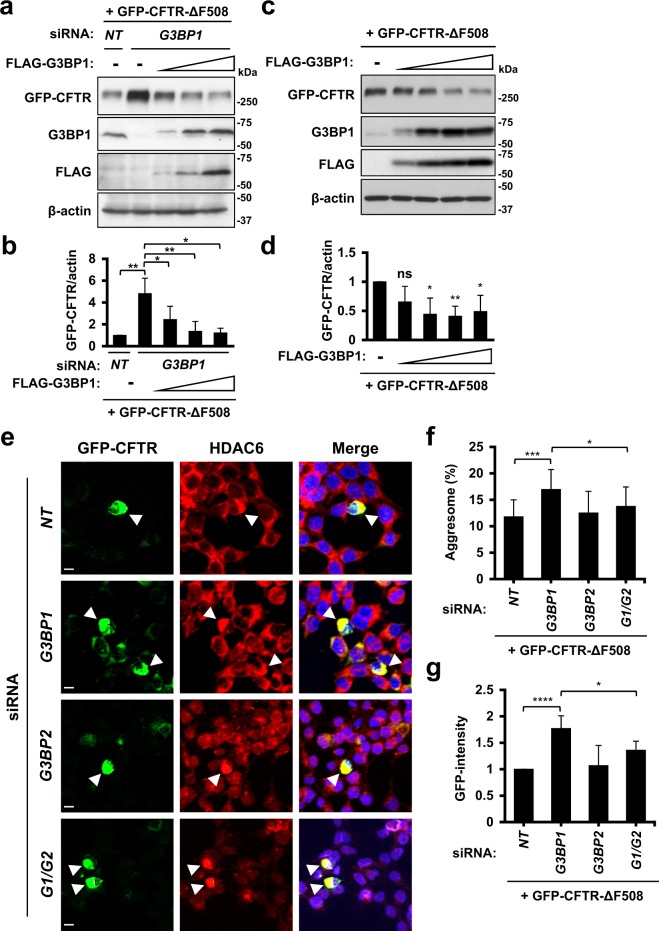
G3BP1-KD induces CFTR-∆F508 aggregation. (**a**,**b**) HeLa cells were transfected with G3BP1-siRNA (*G3BP1*) or control (*NT*), and the cells were then transfected with GFP-CFTR-ΔF508 plasmid along with a sequential amount of FLAG-G3BP1 plasmid (0.0125, 0.025, 0.05 µg). Whole-cell lysates were characterized by a Western blot analysis using anti-GFP (CFTR), anti-G3BP1, anti-FLAG and anti-β-actin antibodies. (**c**,**d**) HeLa cells stably expressing a sequential amount of FLAG-G3BP1 were transfected with GFP-CFTR-ΔF508 plasmid. Whole-cell lysates were subjected to a Western blot analysis using anti-GFP (CFTR), anti-G3BP1, anti-FLAG and anti-β-actin antibodies. The ratios of the GFP-CFTR band to the β-actin one were presented as the means and SD from three experiments in (**b**,**d**). (**e**–**g**) HeLa cells were transfected with G3BP1-siRNA, G3BP2-siRNA, their combination (*G1*/*G2*) or the control (*NT*) by lipofection, and cells were then transfected with the GFP-CFTR-ΔF508 plasmid. The cells were stained with anti-HDAC6 (red) and Hoechst33258 (blue). GFP fluorescence (CFTR), anti-HDAC6 and Hoechst33258 staining was evaluated by a microscope. GFP/HDAC6-double positive aggregates (more than 15 μm^2^ in size) in the perinuclear cytoplasm were considered aggresomes. Arrowheads indicate HDAC6/GFP-CFTR-positive aggresomes. The bars indicate 10 µm. The percentages of cells with GFP/HDAC6-positive aggresomes (Aggresome (%)) were calculated as the ratio of aggresome-positive cells relative to the GFP-positive cells and presented as the mean and SD in (**f**). GFP-intensity was calculated as the ratio of total GFP intensities to control ones (*NT*) in (**g**). *P < 0.05; **P < 0.01; ***P < 0.001; ****P < 0.0001**;** NS: not significant.

To establish the G3BP1-KD activity at the CFTR-ΔF508 protein level, we performed two experiments: a G3BP1 rescue experiment and a G3BP1 overexpression experiment. The exogenous G3BP1 expression in G3BP1-KD cells attenuated the G3BP1-KD-mediated increase in CFTR-ΔF508 protein (Fig. 2a,b). To examine the activity of G3BP1 overexpression at the CFTR-ΔF508 protein level, we established HeLa cells stably expressing a sequential amount of exogenous FLAG-tagged G3BP1 (FLAG-G3BP1) protein via the retrovirus transduction system. Four sequential G3BP1 overexpressions reduced the amount of CFTR-ΔF508 (Fig. 2c,d). These results suggested that the amount of CFTR-ΔF508 protein is reduced by endogenous G3BP1 in HeLa cells and thus increased by G3BP1-KD.

We next examined whether or not G3BP1-KD and G3BP2-KD alter the CFTR-ΔF508 aggregation via immunostaining (Fig. 2e–g). The overexpression of CFTR-ΔF508 in HeLa cells induced CFTR-ΔF508 aggregation and CFTR-ΔF508/HDAC6-double positive aggresome formation^14^. Aggresomes are ubiquitinated protein aggregates induced by the accumulation of ubiquitinated proteins^8,9^. HDAC6 is a marker of aggresomes. Both CFTR-ΔF508 aggregation and CFTR-ΔF508-positive aggresome formation were augmented by G3BP1-KD. While G3BP2-KD hardly affected the aggresome formation of CFTR-ΔF508, it reduced the G3BP1-KD-induced aggresome formation. Two distinct G3BP1-siRNAs augmented the CFTR-ΔF508 aggregation in living Hela and 293T cells (Supplementary Fig. S1).

Taken together, these results suggested that G3BP1 depletion augments CFTR-ΔF508-aggregation and CFTR-ΔF508-positive aggresome formation by increasing the amounts of CFTR-ΔF508 and ubiquitinated proteins, and these augmentations are reduced by G3BP2 depletion.

### G3BP1-KD-induced CFTR-ΔF508 aggregation is mediated by USP10 and p62

**Figure 3:**
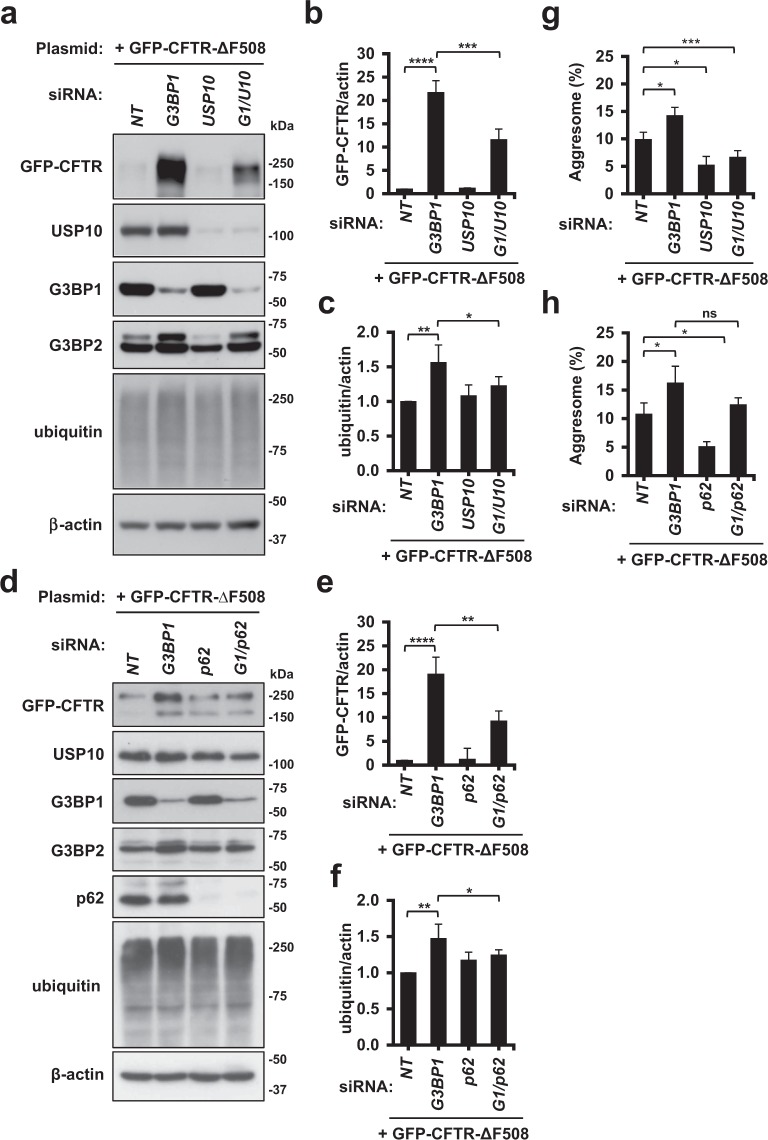
p62 and USP10 mediate G3BP1-KD-induced CFTR-ΔF508 aggregation. (**a**–**f**) HeLa cells were transfected with G3BP1-siRNA (*G3BP1*), USP10-siRNA (*USP10*), p62-siRNA (*p62*), their combination (*G1/U10* or *G1/p62*) or control (*NT*), and cells were then transfected with the GFP-CFTR-ΔF508 plasmid. Whole-cell lysates were subjected to a Western blot analysis using anti-GFP (CFTR), anti-USP10, anti-G3BP1, anti-G3BP2, anti-p62, anti-ubiquitin and anti-β-actin antibodies. The ratios of the GFP-CFTR band or the ubiquitin one to the β-actin one were presented as the means and SD from three experiments in (**b**,**c**,**e**,**f**). (**g**,**h**) HeLa cells were transfected with G3BP1-siRNA (*G3BP1*), USP10-siRNA (*USP10*), p62-siRNA (*p62*), a control (*NT*) or their combination (*G1/U10* or *G1/p62*) by lipofection, and cells were then transfected with the GFP-CFTR-ΔF508 plasmid. The cells were stained with anti-HDAC6 (red) and Hoechst33258 (blue), and GFP fluorescence, anti-HDAC6 and Hoechst33258 staining was evaluated by a microscope. The percentages of cells with GFP/HDAC6-positive aggresomes in the cells (Aggresome (%)) were presented as the mean and SD. *P < 0.05; **P < 0.01; ***P < 0.001; ****P < 0.0001. NS: not significant.

G3BP1 and G3BP2 interact with USP10^20^, and USP10 and its binding partner p62 have been shown to promote ubiquitinated protein aggregation and aggresome formation of CFTR-ΔF508^14^. To do so, p62 interacts with ubiquitinated proteins and augments ubiquitinated protein aggregation. USP10, by interacting with p62, further augments p62-induced ubiquitinated protein aggregation and then stimulates the transport of p62/ubiquitinated protein aggregates to the perinuclear aggresome-forming site. We therefore examined whether or not USP10 and/or p62 plays a key role in G3BP1-KD-induced protein aggregations. A Western blot analysis showed that G3BP1-KD increased the amounts of CFTR-ΔF508 and ubiquitinated proteins, and these increases were reduced by both USP10-KD and p62-KD (Fig. 3a–f). In addition, USP10 overexpression augmented the G3BP1-KD-induced increase in CFTR-ΔF508 protein (Supplementary Fig. S2). Immunofluorescence staining showed that G3BP1-KD increased the amount of CFTR-positive aggresome formation, and this increase was diminished by both USP10-KD and p62-KD (Fig. 3g,h). Taken together, these results suggested that G3BP1 depletion augments the p62/USP10-mediated CFTR-ΔF508 aggregation. It should be noted that the G3BP1-KD-induced G3BP2 increase was also attenuated by p62-KD or USP10-KD. These results further suggested that G3BP1 depletion increases the amount of G3BP2 and ubiquitinated proteins by a similar mechanism.

### G3BP1 and G3BP2 interact with p62

**Figure 4:**
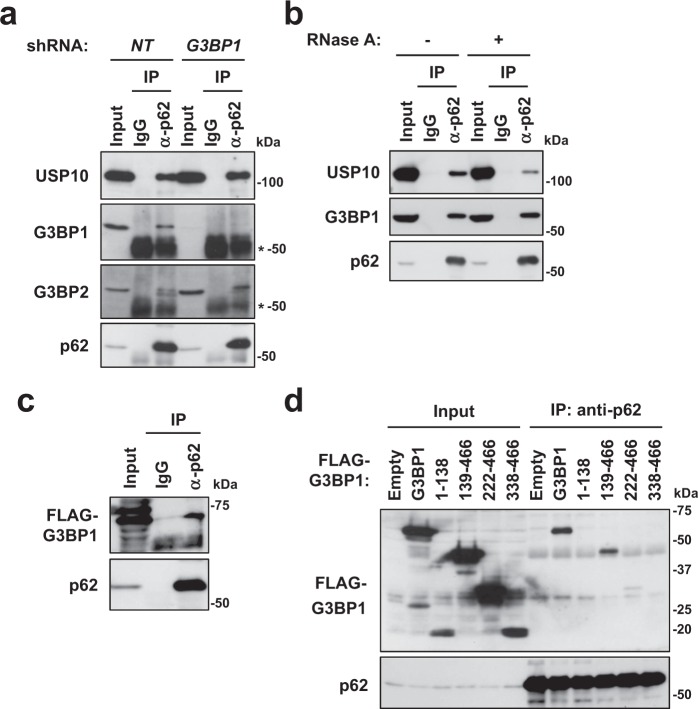
p62 interacts with G3BP1 and G3BP2. (**a**) NP40-soluble lysates were prepared from G3BP1-KD (*G3BP1*) HeLa or its control cells (*NT*) and immunoprecipitated with anti-p62 antibody or normal rabbit IgG. Cell lysate (Input) and immunoprecipitate (IP) were subjected to a Western blot analysis with anti-USP10, anti-G3BP1, anti-G3BP2 and anti-p62 antibodies. Asterisks indicate non-specific bands. (**b**) HeLa cells were lysed with ice-cold NP40-lysis buffer containing RNase A, and soluble cell lysates were immunoprecipitated with anti-p62 antibody or normal rabbit IgG (IgG). The cell lysate (Input) and immunoprecipitate (IP) with or without RNase treatment were subjected to a Western blot analysis with anti-USP10, anti-G3BP1 or anti-p62 antibody. (**c**,**d**) HeLa cells were transfected with FLAG-G3BP1 or its mutant plasmid (G3BP1/1-138, G3BP1/139-466, G3BP1/222-466 or G3BP1/338-466). Cell lysates (Input) and IP with the anti-p62 antibody (IP) were subjected to a Western blot analysis with anti-FLAG (G3BP1) and anti-p62 antibodies.

To elucidate how G3BP1 inhibits p62/USP10-induced protein aggregation, we examined whether or not p62 interacts with G3BP1 and/or G3BP2. An immunoprecipitation analysis showed that p62 interacts with both G3BP1 and G3BP2, and the interaction of p62 with G3BP1 but not G3BP2 or USP10 was reduced in G3BP1-KD cells (Fig. 4a). G3BP1 interacts with many RNA-binding proteins through RNA. While RNase treatment hardly affected the interaction of p62 with G3BP1, it reduced the interaction of p62 with USP10 (Fig. 4b). These results suggested that the interactions of p62 with G3BP1 and USP10 differ in their RNA-dependence. Using G3BP1 mutants, we found that p62 interacted with full-length G3BP1 and weakly with G3BP1/139-466 but not with other deletion mutants (G3BP1/222-466, G3BP1/338-466), suggesting that G3BP1/139-222 has a domain required for interacting with p62 (Fig. 4c,d). G3BP1 and G3BP2 have been shown to interact with USP10^19,20^. Unlike p62, USP10 predominantly interact with the N-terminal portion of G3BP1 and G3BP2^20^. The existence of distinct binding specificities of G3BP1 mutants to p62 and USP10 suggests that G3BP1 and G3BP2 interact with p62 and USP10 by different mechanisms. Taken together, these results suggested that G3BP1 inhibits p62/USP10-induced protein aggregation by interacting with p62 and/or USP10, and interactions of G3BP1 and G3BP2 with p62 and USP10 might explain the antagonistic activities of G3BP1-KD and G3BP2-KD against CFTR-ΔF508 aggregation.

### G3BP1 depletion increases the amount of α-synuclein

**Figure 5:**
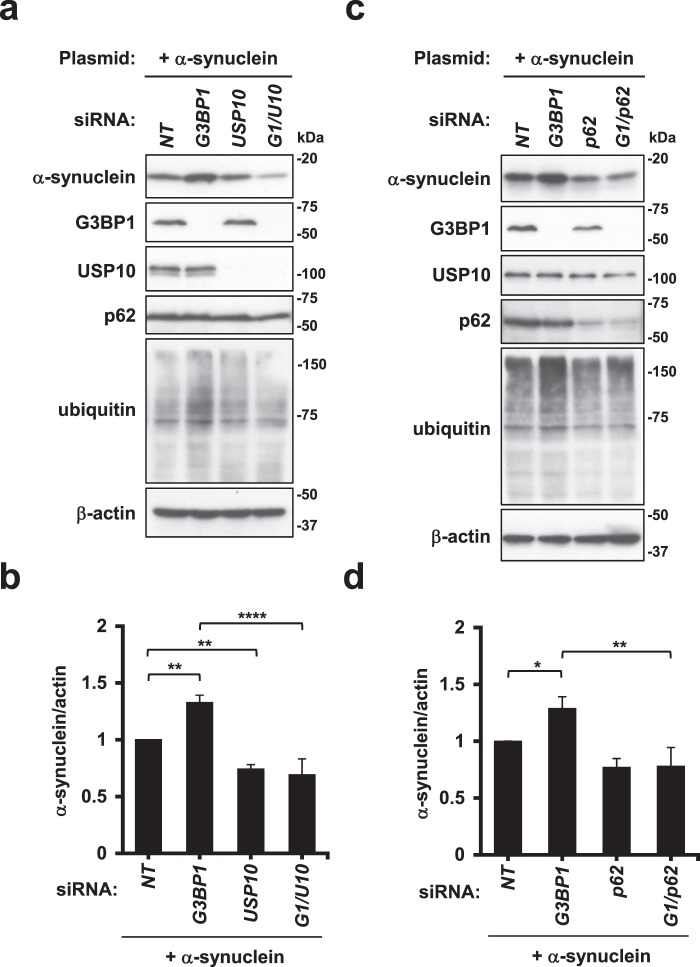
G3BP1-KD increase the amount of α-synuclein. (**a**–**d**) HeLa cells were transfected with G3BP1-siRNA (*G3BP1*), USP10-siRNA (*USP10*), p62-siRNA (*p62*), their combination (*G1/U10* or *G1/p62*) or a control (*NT*), and then cells were transfected with the α-synuclein plasmid. Whole-cell lysates were subjected to a Western blot analysis using anti-α-synuclein, anti-G3BP1, anti-USP10, anti-p62, anti-ubiquitin and anti-β-actin antibodies. The ratios of the α-synuclein band to the β-actin one were presented as the means and SD from three experiments in (**b**,**d**). *P < 0.05; **P < 0.01; ****P < 0.0001.

α-synuclein is a causative factor of PD, and the presence of α-synuclein aggregates in the neurons of brain lesions of PD patients, called Lewy bodies, is a hallmark pathology of PD^25,26^. Like CFTR-ΔF508, USP10 and p62 have been shown to promote the aggregation of α-synuclein protein by increasing the amount of protein^14^. We therefore examined whether or not G3BP1 regulates α-synuclein aggregation (Fig. 5). A Western blot analysis detected α-synuclein in HeLa cells, and the amount of α-synuclein was increased by G3BP1-KD. Like CFTR-ΔF508, the G3BP1-KD-induced increase in α-synuclein protein was reduced by both USP10-KD and p62-KD. These results suggested that G3BP1 depletion increases the amount of α-synuclein protein in USP10- and p62-dependent manners.

### G3BP1 depletion increases the amounts of ubiquitinated CFTR-ΔF508 and α-synuclein

**Figure 6:**
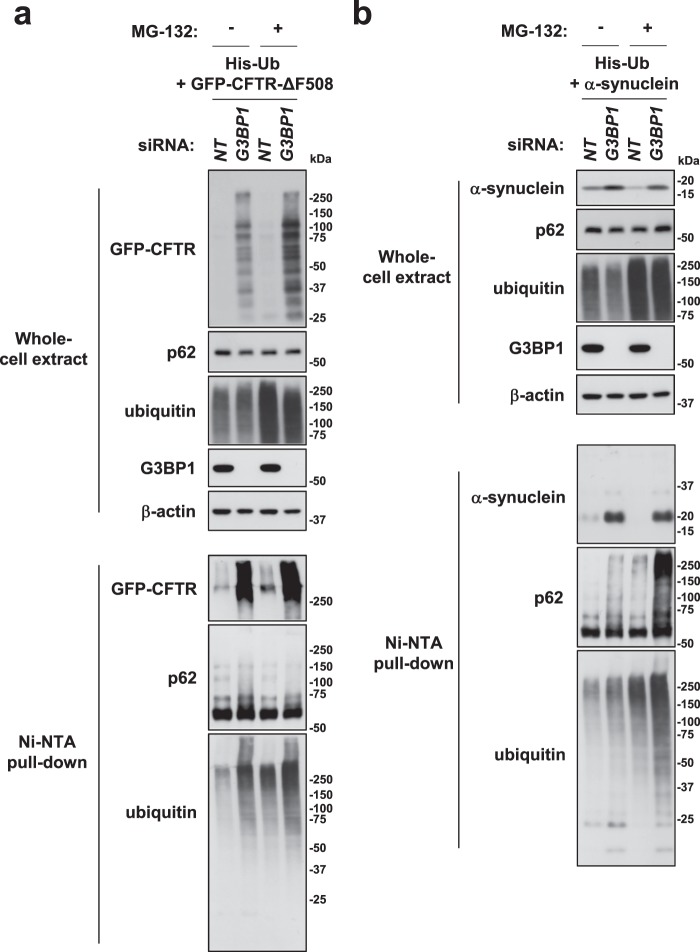
G3BP1-KD induces the ubiquitination of CFTR-∆F508 and α-synuclein. (**a**,**b**) HeLa cells were transfected with either G3BP1-siRNA (*G3BP1*) or control siRNA (*NT*) by Lipofectamine RNAiMAX. Cells were further transfected with the His-ubiquitin plasmid either with GFP-CFTR-ΔF508 (**a**) or α-synuclein plasmid (**b**). Cells were then treated with 5 µM MG-132 or DMSO for 6 h. Cells were lysed with buffer A, and the lysates were incubated with Ni-NTA-agarose at room temperature for 3 h. His-ubiquitinated proteins bound to the Ni-NTA agarose were characterized by a Western blot analysis.

The amounts of CFTR-ΔF508 and α-synuclein protein are regulated by ubiquitination-induced degradation by ubiquitin-proteasome system and/or autophagy^27,28^. We therefore measured whether or not G3BP1-KD increases the amounts of ubiquitination of CFTR-ΔF508 and/or α-synuclein in HeLa cells by transfecting the six-histidine-tagged ubiquitin (His-ubiquitin) (Fig. 6). An ubiquitination assay using His-ubiquitin uses 6 M guanidine for cell lysis. Therefore, this assay can detect NP40-soluble and NP40-insoluble ubiquitinated proteins. A pull-down of His-ubiquitins detected a low amount of ubiquitinated GFP-CFTR-ΔF508 in G3BP1-competent (G3BP1-WT) cells, but the amount was increased in G3BP1-KD cells (Fig. 6a). MG-132 treatment also increased the amount of ubiquitinated CFTR-ΔF508. In addition, G3BP1-KD increased the amount of His-ubiquitinated proteins with and without MG-132 treatment. Taken together, these results suggest that G3BP1-KD increases the amount of ubiquitinated CFTR-ΔF508, partly through inhibition of proteasome activity. It should be noted that His-ubiquitin overexpression induced multiple low-molecular-weight CFTR-ΔF508 proteins in G3BP1-KD cells, possibly through His-ubiquitin-induced ubiquitination and partial degradation of CFTR-ΔF508. In addition, the amount of ubiquitinated α-synuclein protein increased by G3BP1-KD, although it was hardly affected by MG-132 treatment (Fig. 6b). In contrast to CFTR-ΔF508, we detected predominantly mono-ubiquitinated α-synuclein protein, based on the molecular weight. This is consistent with the findings of previous studies that α-synuclein is predominantly mono-ubiquitinated in cells^28^. In addition, G3BP1-KD increased the amount of ubiquitinated p62 in the presence of GFP-CFTR-ΔF508 or α-synuclein (Fig. 6a,b). Given that ubiquitination of p62 augments the interaction with ubiquitinated proteins, these results suggest that G3BP1-KD in the presence of an ubiquitination-prone protein, such as GFP-CFTR-ΔF508 or α-synuclein, augments the interaction of p62 with ubiquitinated proteins.

We also examined ubiquitinated GFP-CFTR-ΔF508 in the NP40-soluble fraction using anti-GFP immunoprecipitation (Supplementary Fig. S3). An anti-GFP detected a low amount of ubiquitinated GFP-CFTR-ΔF508 in G3BP1-WT cells, but the amount was increased in G3BP1-KD cells. MG-132 treatment prominently increased the amount of ubiquitinated CFTR-ΔF508, and the amount was further increased by G3BP1-KD. These results further suggested that G3BP1-KD increases the amount of ubiquitinated CFTR-ΔF508. In addition, G3BP1-KD with MG-132-treatment increased the binding of CFTR-ΔF508 to p62. Given that p62 interacts with ubiquitinated proteins, these results suggested that G3BP1-KD augments the binding of CFTR-ΔF508 with p62 by increasing the amount of ubiquitinated CFTR-ΔF508.

### G3BP1 depletion inhibits proteasome activity

**Figure 7:**
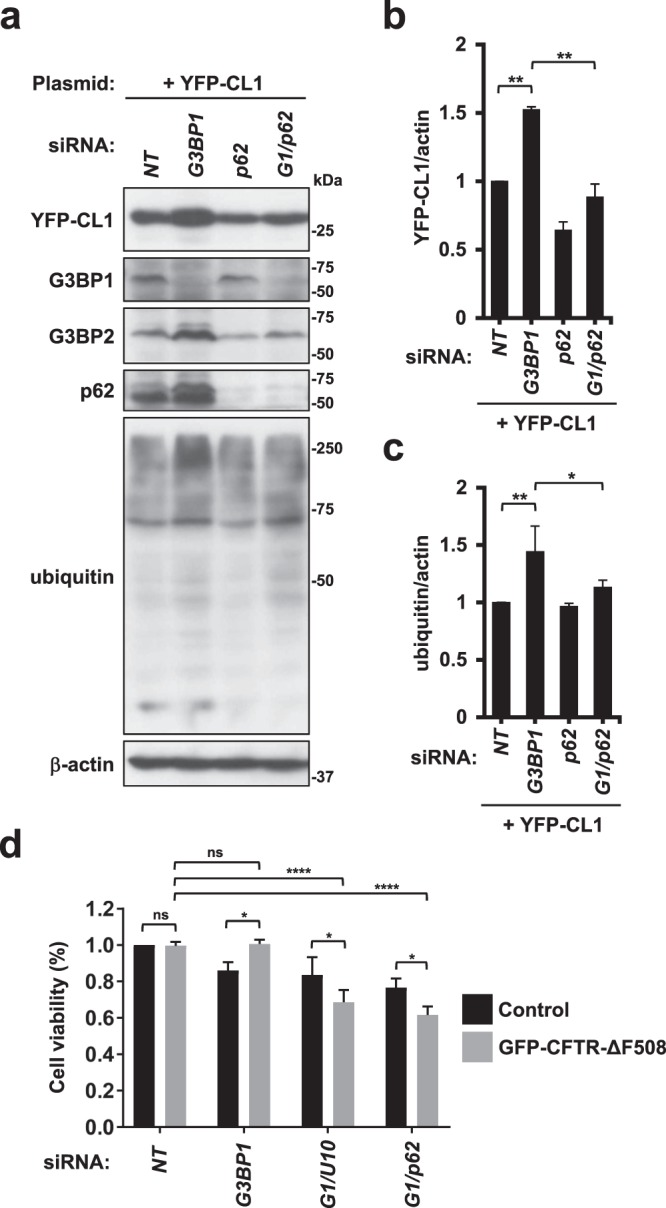
G3BP1 inhibits proteasome activity. (**a**–**c**) HeLa cells were transfected with G3BP1-siRNA (*G3BP1*), p62-siRNA (*p62*), their combination (*G1/p62*) or the control (*NT*) and then transfected with the YFP-CL1 plasmid. Whole-cell lysates were subjected to a Western blot analysis using anti-GFP (YFP-CL1), anti-G3BP1, anti-G3BP2, anti-p62, anti-ubiquitin or anti-β-actin antibodies. The ratios of the YFP-CL1 band or the ubiquitin one to the β-actin one were presented as the means and SD from three experiments in (**b**,**c**). (**d**) G3BP1, USP10 and p62 control cytotoxicity of GFP-CFTR-ΔF508. HeLa cells were transfected with G3BP1-siRNA (*G3BP1*), G3BP1-siRNA plus USP10-siRNA (*G1*/*U10*), G3BP1-siRNA plus p62-siRNA (*G1*/*p62*) or control (*NT*) and then further transfected with the GFP-CFTR-ΔF508 plasmid. To measure the metabolic activity of cells (Cell viability) by Cell Counting Kit-8 (CCK-8), cells were treated with CCK-8 solution for 1 h, and the absorbance (450 nm) of culture medium was measured by TriStar LB 941 Microplate Reader. Absorbance was normalized to the value of cells transfected with control siRNA (*NT*) (without GFP-CFTR∆F508 plasmid), and the ratio was presented as the mean and SD from three experiments. *P < 0.05; **P < 0.01; ****P < 0.0001. NS: not significant.

G3BP1-KD increased the amount of ubiquitinated proteins, including CFTR-ΔF508. These results suggest that G3BP1-KD inhibits the proteasome-mediated degradation of ubiquitinated proteins. To explore this possibility, we used the proteasome reporter YFP-CL1^29^. YFP-CL1 is a fusion protein of yellow fluorescent protein (YFP) with a 16-amino-acid peptide containing a ubiquitination site. Therefore, YFP-CL1 protein is degraded by the ubiquitin-proteasome system and its degradation is inhibited by proteasome inhibitor treatment. G3BP1-KD increased the amount of YFP-CL1 protein, and this increase was reduced by p62-KD (Fig. 7a–c). These results suggested that G3BP1 depletion inhibits the proteasome activity, thereby increasing the amount of ubiquitinated proteins, including CFTR-ΔF508, and this inhibition is mediated by p62.

### USP10-KD and p62-KD augments cell toxicity of CFTR-ΔF508

Several ubiquitinated protein aggregates, including α-synuclein, especially their oligomers, have been shown to exhibit cell toxicity and then reduce cell viability^30^. We next examined whether or not ubiquitination and aggregation of CFTR-ΔF508 regulated by G3BP1, p62 and USP10 affect the CFTR-ΔF508-induced cell toxicity by measuring the metabolic activity of HeLa cells using the Cell Counting Kit-8 (CCK-8) (Fig. 7d). G3BP1-KD only slightly affected the cell viability with GFP-CFTR-ΔF508 expression, but G3BP-1-KD together with USP10-KD or p62-KD markedly reduced the cell viability. Both G3BP1-KD/USP10-KD and G3BP1-KD/p62-KD exerted cell toxicity without GFP-CFTR-ΔF508, but the level was less than that with GFP-CFTR-ΔF508. We showed that G3BP1-KD augments the ubiquitination and aggregation of CFTR-ΔF508, and the augmented aggregation is attenuated by both USP10-KD and p62-KD (Figs 2 and 6). Taken together, these results suggest that the G3BP1-KD-induced toxicity of ubiquitinated CFTR-ΔF508 was masked by the G3BP1-KD-induced CFTR-ΔF508 aggregation, but the toxicity of ubiquitinated CFTR-ΔF508 was unmasked by a reduction of the ubiquitinated CFTR-ΔF508 aggregation induced by both USP10-KD and p62-KD.

### A low expression of G3BP1 in PD and non-PD brains

**Figure 8:**
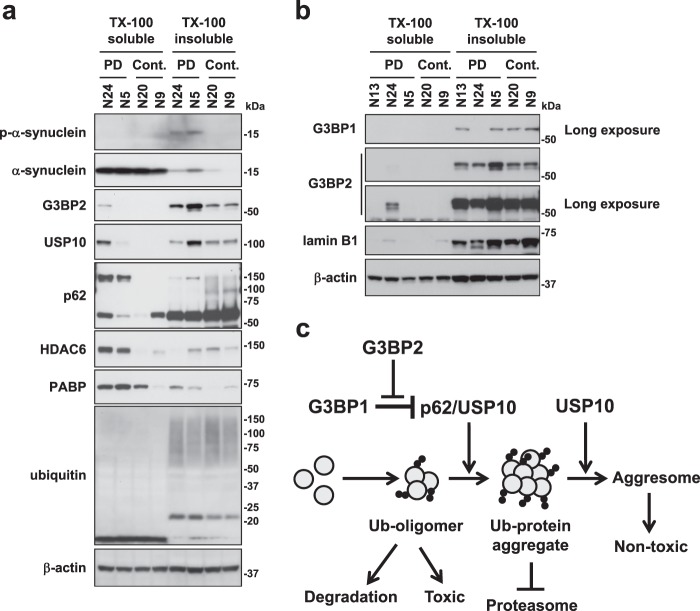
The expression of aggregation-associated proteins in brain lesions in PD patients. (**a**,**b**) Detergent-soluble and detergent-insoluble lysates were prepared from brain tissues (amygdala) of PD patients (PD) and controls (Cont.). These cell lysates were subjected to a Western blot analysis using their respective antibodies. P-α-synuclein indicates phosphorylated α-synuclein. (**c**) A schematic model of G3BP1 and G3BP2 activities on protein aggregation. p62 and USP10 promote protein aggregation, and the induced protein aggregates inhibit the proteasome activity to further augment protein aggregation. G3BP1 inhibits p62/USP10-induced protein aggregation, and this inhibition is attenuated by G3BP2. Ubiquitinated protein oligomers are toxic, but the toxicity was attenuated by aggresome formation.

To gain insight into how G3BP1 regulates α-synuclein aggregation in PD patients, we examined the expression of G3BP1 and other aggregation-associated proteins in the brain tissue (amygdala) of PD patients and non-PD individuals after fractionating the brain lysates into detergent-soluble and detergent-insoluble fractions (Fig. 8a, Table 1). A Western blot analysis detected that PD patients expressed increased amounts of soluble and/or insoluble USP10, p62, G3BP2, poly(A)-binding protein (PABP) and HDAC6 relative to the controls (Fig. 8a). Like USP10, HDAC6 augments aggresome formation in cultured cells^13^ and is localized in Lewy bodies. PABP is a binding protein of USP10, G3BP1 and G3BP2. These results supported the proposed notion that USP10, p62 and HDAC6 play a role in α-synuclein aggregation and Lewy body formation through aggresome-related mechanism^14,31^. We detected a soluble p62-multimer only in PD brain, not non-PD brain. Given that p62 multimer is an activated form^32^, these results suggest that p62 multimer in PD brain stimulates α-synuclein aggregation.

**Table 1:** Clinicopathologic profiles of the patients.

	Age at death (yrs)	Sex	Clinical Diagnosis	Pathological Diagnosis
**Control**				
N20	79	F	Spontaneous CSF leaks	Multiple microinfarcts
N9	90	M	Myopathy	Myopathy
**PD**				
N13	79	M	PD	PD (DLB neocortical type)
N24	87	F	PD	PD (DLB neocortical type)
N5	80	M	PD	PD (DLB neocortical type)

PD: Parkinson’s disease; DLB: Dementia with Lewy bodies; CSF: cerebrospinal fluid.

In contrast, two of three PD and two non-PD samples expressed a low amount of G3BP1 protein relative to those of G3BP2, p62 and USP10, and intriguingly, G3BP1 protein was undetectable in one PD patient (N24) (Fig. 8a,b). These results suggest that a low or reduced expression of G3BP1 in PD brains promotes p62/USP10-induced protein aggregation. However, we did not observe marked differences in the expression of ubiquitin between PD patients and controls, suggesting that the amount of ubiquitinated proteins is differently regulated from that of other aggregation-associated proteins characterized here in PD patients.

## Discussion

The aberrant accumulation of ubiquitinated protein aggregates in cells is the hallmark pathology of many degenerative diseases, including PD and CF^1–3^. To prevent devastating outcomes due to the aggregation of ubiquitinated protein, cells must reduce the amount of ubiquitinated proteins by either degrading them or inactivating their toxicities. In this study, we found that G3BP1 inhibits protein aggregation and aggresome formation by reducing the amount of ubiquitinated proteins in the steady state condition of cultured cells, and these activities target two pathogenic proteins: CFTR-ΔF508 and α-synuclein (Fig. 8c). Therefore, the present study suggested that G3BP1 is a negative regulator of protein aggregation, and its reduction or dysfunction promotes pathological protein aggregation in degenerative disorders, including CF and PD.

USP10 and p62 have been shown to induce protein aggregation and aggresome formation^14^. We found that G3BP1 depletion stimulates p62/USP10-induced protein aggregation and aggresome formation, and the stimulation is attenuated by G3BP2 depletion (Figs 2, 3 and 8c). Both G3BP1 and G3BP2 interacted with p62 and USP10^20^ (Fig. 4). Taken together, these results suggested that G3BP1 inhibits p62/USP10-induced protein aggregation, whereas G3BP2 attenuates such a G3BP1 inhibition by competing G3BP1-interaction with p62 and/or USP10 (Fig. 8c).

While G3BP1 and G3BP2 are structurally and functionally related, they antagonistically regulated protein aggregation (Figs 1 and 2). For instance, G3BP1-KD-induced CFTR-ΔF508 aggregation was reduced by adding G3BP2-KD (Figs 1c–e and 2e–g). It should be noted that G3BP1 and G3BP2 have several differences. While G3BP2 is a ubiquitinated protein and degraded by the ubiquitin-proteasome system, it can be deubiquitinated by USP10^24^. In contrast, while ubiquitination of G3BP1 has not been reported, G3BP1 inhibits the deubiquitinase activity of USP10^19^. These differences between G3BP1 and G3BP2 may therefore play a role in their distinct activities concerning protein aggregation.

A proteasome reporter and the findings of the G3BP-1-KD experiments suggest that G3BP1 depletion inhibits both the proteasome activity and the degradation of ubiquitinated proteins, including CFTR-ΔF508 (Figs 1 and 7). Accumulating evidence show that protein aggregates inhibit proteasome activity, which further increases the amount of ubiquitinated proteins and protein aggregates^33^. p62 and USP10 have been shown to inhibit the proteasome activity by promoting ubiquitinated protein aggregation^14^. Taken together, these results suggest that G3BP1 depletion inhibits proteasome activity by promoting p62/USP10-induced protein aggregation.

G3BP1 depletion in HeLa cells induced α-synuclein ubiquitination by a proteasome-independent mechanism (Fig. 6b). This G3BP1-KD-induced α-synuclein ubiquitination might be explained by two mechanisms. Given that ubiquitinated α-synuclein has been shown to be degraded by autophagy, G3BP1 depletion might inhibit autophagy in order to reduce degradation of ubiquitinated α-synuclein. Alternatively, G3BP1 depletion might modulate the activity of either α-synuclein ubiquitinase or deubiqutinase in order to augment α-synuclein ubiquitination. Further analyses will be required in order to understand how G3BP1 inhibits α-synuclein ubiquitination and aggregation.

USP10 and p62 have been shown to inhibit ubiquitinated protein-induced apoptosis by MG-132 treatment through inducing ubiquitinated protein aggregation and aggresome^14^. Evidence suggests that while ubiquitinated protein oligomers have cell toxicity, such toxicity is attenuated by forming ubiquitinated protein aggregates (aggresomes)^30^. The present findings support this notion, as follows (Fig. 7d). Ubiquitinated CFTR-ΔF508 by itself did not show cell toxicity, since ubiquitinated CFTR-ΔF508 is rapidly degraded by proteasome. G3BP1-KD increased the amount of ubiquitinated CFTR-ΔF508 but not the toxicity, since G3BP1-KD simultaneously induced CFTR-ΔF508 aggregation (aggresome). However, p62-KD or USP10-KD restored CFTR-ΔF508 toxicity in G3BP1-KD cells by reducing CFTR-ΔF508 aggregation. Further studies will be required in order to clarify the roles of G3BP1, USP10 and p62 in cell toxicity as well as protein ubiquitination and aggregation.

G3BP1 depletion increased the amounts of ubiquitinated proteins other than α-synuclein and CFTR-ΔF508 (Figs 1 and 7). First, a proteasome reporter suggested that G3BP1 depletion inhibits proteasome activity (Fig. 7). Second, G3BP1 depletion increased the amounts of multiple ubiquitinated proteins with different molecular weights (Fig. 1). G3BP1 has been shown to play critical roles in innate immunity, tumor development and RNA metabolism^20,34^. Thus, G3BP1 might play these roles through changes in the ubiquitination status of target proteins in certain contexts.

We detected a low amount of G3BP1 protein relative to G3BP2, p62 and USP10 in PD and non-PD brains (amygdala) (Fig. 8). Kennedy *et al*. detected only G3BP2 but not G3BP1 protein in adult mouse brain lysates by a Western blot analysis^35^. *In situ* hybridization analyses using antisense RNA also showed that the amount of G3BP1 mRNA in almost the all of the mouse brain regions is lower than those of other tissues^36^. These results suggested that p62 and USP10 in brain cells promote ubiquitinated protein aggregation more efficiently than non-brain cells with a relatively high G3BP1 expression. It should be noted that G3BP1-knockout mice develop neurodegeneration with neuronal dysfunction and neuronal apoptosis^23^. These results suggest that despite its low expression, G3BP1 still plays a protective role in the neuronal survival and development of neurodegeneration. It is worth noting that one PD patient (N24) expressed an undetectable amount of G3BP1, which might have played a key role in the α-synuclein ubiquitination and aggregation in this patient. In addition, given that G3BP2 reduces the G3BP1-mediated inhibition of p62/USP10-induced protein aggregation, increased G3BP2 expression in PD brain might augment p62/USP10-induced protein aggregation. Further analyses will be required in order to elucidate how G3BP1 and G3BP2 regulate protein aggregation in neurodegenerative diseases, including PD.

## Methods

### Cell lines and culture condition

HeLa, 293T and Plat-E cells were cultured in Dulbecco’s modified Eagle’s medium (DMEM) supplemented with 10% heat-inactivated fetal bovine serum (FBS), 4 mM L-glutamine, 50 units/ml penicillin, 50 µg/ml streptomycin and MEM non-essential amino acid solution (Thermo Fisher Scientific, Waltham, MA, USA).

### Reagents and antibodies

The following reagents were purchased from the indicated companies: MG-132 (474790; Calbiochem, Danvers, MA, USA) and Hoechst 33258 (H-3569; Molecular Probes, Eugene, OR, USA). The following antibodies were used in this study: anti-USP10 (A300-901A; Bethyl Laboratories, Montgomery, TX, USA; HPA006731; Sigma-Aldrich, St. Louis, MO, USA), anti-ubiquitin (sc-8017; Santa Cruz Biotechnology, Santa Cruz, CA, USA), anti-p62 (PM045; MBL, Nagoya, Japan, GP62-C; PROGEN, Heidelberg, Germany), anti-G3BP1 (611127; BD Transduction Laboratories, San Jose, CA), anti-G3BP2 (A302-040; Bethyl Laboratories), anti-PABP (ab21060; Abcam, Cambridge, GB), anti-HDAC6 (sc-11420; Santa Cruz Biotechnology), anti-FLAG (M2 Monoclonal Antibody; Sigma-Aldrich), anti-GFP (sc-9996; Santa Cruz Biotechnology), anti-lamin B1 (sc-374015; Santa Cruz Biotechnology), anti-α-synuclein (S5566; Sigma-Aldrich), anti-phosphorylated α-synuclein (015-25191; FUJIFILM Wako Pure Chemical Corporation, Osaka, Japan), anti-β-actin (sc-47778; Santa Cruz Biotechnology), anti-HA (2367S; Cell Signaling, Beverly, MA, USA) and anti-α-tubulin (CP06 Oncogene Research Products, Boston, MA, USA).

### Plasmids

FLAG-tagged G3BP1 expression plasmid (pFLAG-G3BP1) and its mutants were described previously^20^. The pMXs-FLAG-G3BP1-Puro was a G3BP1 retroviral vector plasmid constructed by inserting a FLAG-G3BP1 DNA sequence prepared from the pFLAG-G3BP1 plasmid by polymerase chain reaction into a multicloning site of the pMXs-Puro retroviral vector (Cell Biolabs, Inc., San Diego, CA, USA). The HA-tagged USP10 (HA-USP10) expression plasmid was described previously^14^. pMD.G is the expression vector of the envelope glycoprotein (G protein) of vesicular stomatitis virus and a kind gift from Dr. Didier Trono (Swiss Federal Institute of Technology in Lausanne, Switzerland). GFP-CFTR-ΔF508 plasmid was a gift from Dr. Ron Kopito (Stanford University, Palo Alto, CA, USA). α-synuclein plasmid was obtained from Dr. Masato Hasegawa (Tokyo Metropolitan Institute of Medical Science, Tokyo, Japan). YFP-CL1 plasmid was a gift from Nico Dantuma. The six-His-ubiquitin plasmid was provided by Dr. Dirk Bohmann (University of Rochester Medical Center, Rochester, NY, USA).

### Plasmid transfection

HeLa cells (1.5 × 10^5^) were seeded onto a 6-well plate (Corning, NY, USA) the day before transfection, and cells were transfected with the plasmid by FuGENE 6 in Opti-MEM (1869048; Life Technologies, Carlsbad, CA) according to the manufacturer’s (Roche, Basel, Switzerland). Cells were harvested for analysis at 24 h after transfection.

### RNA interference

Cells were transfected with small interfering RNA (siRNA) (20–100 pmol) using Lipofectamine RNAiMAX reagents according to the manufacturer’s protocol (Invitrogen, Carlsbad, CA, USA). The following siRNAs were purchased from the companies indicated: G3BP1-siRNA specific to the 3′ untranslated region of human G3BP1 (oligo identification number [OID]: SASI_Hs01_00045804) and its negative control (MISSION_siRNA Universal Negative Control #1) were purchased from Sigma-Aldrich; another set of human G3BP1-siRNAs (OID:1027261) and its negative control siRNA (OID:02665194) were purchased from QIAGEN (Hilden, Germany); and human p62-siRNAs (OID: HSS113116, HSS113117), human G3BP2-siRNA (OID: HSS114988), human USP10-siRNA (OID: HSS113448) and their negative control siRNA (Cat. No. 12935-100) were purchased from Invitrogen.

### Stable-knockdown of G3BP1

Stable knockdown of endogenous G3BP1 in HeLa cells was carried out using the lentivirus vector pLKO.1-puro encoding G3BP1-shRNA (Sigma-Aldrich). HIV-1-based lentivirus encoding G3BP1-shRNA was produced by cotransfection of the three plasmids (pLKO.1-puro-G3BP1-shRNA: 4.28 μg; pCAG-HIVgp: 2.86 μg; pCMV-VSV-G-RSV-Rev: 2.86 μg) into 293 T cells (2 × 10^6^) using the FuGENE HD reagent according to the manufacturer’s instructions (Promega, Madison, WI), and HeLa cells were infected with the virus in the presence of 8 μg/μl polybrene. These cells were cultured in selection medium containing 2 μg/ml puromycin.

### Western blot analyses

Cells were lysed with sodium dodecyl sulfate (SDS) lysis buffer (62.5 mM Tris-HCl, pH 6.8, 2% SDS, 10% glycerol, 5% 2-mercaptoethanol and 0.005% bromophenol blue), and cell lysates (20 μg) were separated by SDS-PAGE and electrophoretically transferred onto a PVDF membrane (Immobilon; Millipore). PVDF membranes were incubated with 5% skimmed milk and further treated with the indicated antibodies diluted in Can Get (TOYOBO, Osaka, Japan). Immunoreactive bands were detected with an enhanced chemiluminescence (ECL) detection system (ECL Western Blotting Detection Reagents; GE Healthcare, Chicago, IL, USA; Pierce^TM^ ECL Plus Western Blotting Substrate; Thermo Fisher Scientific) and visualized using Amersham Hyperfilm ECL (GE Healthcare). For the detection of α-synuclein protein, the PVDF membrane was fixed with 0.4% paraformaldehyde before incubating with skimmed milk, since α-synuclein protein is prone to separate from the membrane^37^.

### Cell fractionation

Whole-cell lysate, NP40-soluble lysate and NP40-insoluble lysate were prepared as follows: cells were lysed with cold NP40-lysis buffer (1% Nonidet P-40, 25 mM Tris-HCl, pH 7.2, 150 mM NaCl, 1 mM EDTA, 1 mM phenylmethylsulfonyl fluoride, 20 μg/ml aprotinin). After centrifugation of cell lysates, the supernatant was used as the NP40-soluble lysate. The resultant pellet was treated with SDS-lysis buffer and then sonicated. After centrifugation, the supernatant was used as the NP40-insoluble lysate. To prepare whole-cell lysate, cells were directly treated with the SDS-lysis buffer and then sonicated. After centrifugation, the supernatant was used as the whole-cell lysate. These three lysates were subjected to a Western blot analysis.

Autopsy brain samples from PD patients and controls were treated with ice-cold Triton X-100 lysis buffer (1% Triton X-100, 50 mM Tris-HCl, pH 7.5, 150 mM NaCl, 1 mM phenylmethylsulfonyl fluoride, 20 μg/ml aprotinin), and the Triton X-100-soluble and Triton X-100-insoluble lysates were collected by a method similar to that described above. The two lysates were then subjected to a Western blot analysis.

The study using human samples was performed with the approval of the ethics committees of Niigata University (approval number: G2015-0686). Written informed consent for an autopsy, collection of samples and the subsequent use of the samples for research purposes was obtained from the next of kin of the deceased persons involved in this study. All experiments were performed in accordance with relevant guidelines and regulations.

### Immunoprecipitation assay

Cells cultured in a 6-well plate (Corning) were lysed with ice-cold NP40 lysis buffer. Soluble cell lysates were immunoprecipitated by the primary antibody, and immune complexes were precipitated by protein G-sepharose beads (GE Healthcare). The beads were washed and boiled in SDS-lysis buffer, and then the proteins released from the beads were subjected to a Western blot analysis. To assess the RNA-dependent interaction, cells were lysed with ice-cold NP40-lysis buffer containing RNase A (Nippon Gene, Tokyo, Japan) at a final concentration of 100 μg/mL.

### Establishment of HeLa cell lines stably expressing G3BP1 protein

To prepare retroviruses expressing G3BP1, 2 × 10^5^ Plat-E cells (Cell Biolabs, Inc.) were seeded onto a 60-mm dish and co-transfected with 0.3 µg pMD.G with either 1.2 µg pMXs-FLAG-G3BP1-Puro or pMXs-Puro using FuGENE6. At 24 h after transfection, the culture media was replaced with fresh media. After a further 24 h culture, the viruses in the culture media were filtered through a membrane with a 0.45-µm pore size. For virus infection, 1 × 10^5^ HeLa cells were seeded onto a 6-well plate and infected with 500 µL of pMXs-Puro retrovirus or 250, 500, 750 or 1000 µL of pMXs-FLAG-G3BP1-Puro retrovirus in the presence of 8 µg/mL polybrene for 24 h. Infected cells were cultured with 2 µg/mL puromycin in fresh media for 48 h.

### Quantification of ubiquitinated protein

HeLa cells (2 × 10^5^) were cultured on 6-cm dishes (Corning) and transfected with either G3BP1-siRNA or the control siRNA (*NT*) by Lipofectamine RNAiMAX. Cells were further transfected with the six-His-ubiquitin plasmid with either GFP-CFTR-ΔF508 or α-synuclein plasmid by FuGENE 6. Cells were then treated with 5 µM MG-132 or DMSO for 6 h and lysed with buffer A (6 M guanidine-HCl, 0.1 M Na_2_HPO_4_, 0.1 M NaH2PO4, 10 mM imidazole, pH 8.0). Cell lysates were incubated with Ni-NTA-agarose (QIAGEN) at room temperature for 3 h. The Ni-NTA agarose was then washed 3 times with buffer A, 2 times with buffer A plus buffer B (1:4), and 2 times with buffer B (25 mM Tris-HCl pH 6.8, 20 mM imidazole). Proteins bound to the Ni-NTA agarose were then eluted by SDS-lysis buffer containing 200 mM imidazole. The eluted proteins were subjected to a Western blot analysis.

### Immunofluorescence analyses

Cells were plated on glass coverslips in a 6-well plate followed by fixing with 3.7% formaldehyde in phosphate-buffered saline (PBS) at room temperature for 15 min and then permeabilized by 0.1% Triton X-100 in PBS at room temperature for 5 min. Fixed cells were incubated with the primary antibody at room temperature for 60 min, washed with PBS, and then incubated with the secondary antibody and Hoechst 33258 for nuclear staining at room temperature for 60 min. Staining of cells was evaluated using a fluorescence microscope (BZ-8000; KEYENCE, Osaka, Japan) using a fluorescent analysis software package (BZ-II analyzer; KEYENCE, Osaka, Japan). The secondary antibodies used were anti-mouse immunoglobulin labelled with either Alexa488 or Alexa594, or anti-rabbit immunoglobulin labelled with either Alexa488 or Alexa594 (Molecular Probes).

To measure cells with aggresomes induced by GFP-CFTR-ΔF508, more than 300 cells from 3 or 4 coverslips were evaluated. One large GFP/HDAC6-positive aggregate (more than 15 μm^2^ in size) at the perinuclear cytoplasmic region was evaluated as an aggresome. Percentages of cells with GFP/HDAC6-positive aggresomes were presented as the ratio of aggresome-positive cells relative to the GFP-positive cells. The expression of GFP-CFTR-ΔF508 in the cells was measured using a fluorescent analysis software package (BZ-II analyzer), and GFP-intensity was presented as the ratio of total GFP intensities in in more than 300 cells in 10 random fields from 3 or 4 coverslips. Cells numbers were counted by Hoechst staining.

### Cell viability assay

Cell viability was measured by Cell Counting Kit-8 (CCK-8) (DOJINDO, Kumamoto, Japan). HeLa cells cultured on 24-well plate were transfected with siRNA (50 pmol) using Lipofectamine RNAiMAX and then further transfected with the GFP-CFTR-ΔF508 plasmid. Cells were then treated with CCK-8 solution containing WST-8 for 1 h, and the absorbance (450 nm) of culture medium was measured by TriStar LB 941 Microplate Reader (Berthold, Bad Wildbad, Germany).

### Statistical analyses

Statistical analyses were performed using one-way or two-way ANOVA with the Prism7 software program (GraphPad, San Diego, CA). The data were presented as the mean and standard deviation (SD).

## Supplementary information

Supplementary Information

## Data Availability

The data that support the findings of this study are available from the corresponding author upon reasonable request.
