# Supplementary material for: G3BP1 inhibits ubiquitinated protein aggregations induced by p62 and USP10
Source: Sci Rep. 2019 Sep 9;9:12896. doi: 10.1038/s41598-019-46237-1 (PMC6733845; doi:10.1038/s41598-019-46237-1)
Supplement: Supplementary file 1 — Supplementary Information [file 41598_2019_46237_MOESM1_ESM.pdf]

Supplementary Information

G3BP1 inhibits the ubiquitinated protein aggregations induced by p62 and USP10

Sergei Anisimov,[1,*] Masahiko Takahashi,[1,*] Taichi Kakihana,[1] Yoshinori Katsuragi,[1] Hiroki Kitaura,[2] Lu Zhang,[2] Akiyoshi Kakita,[2] Masahiro Fujii[1]

[1]Division of Virology, Niigata University Graduate School of Medical and Dental Sciences, Niigata, 951-8510, Japan
[2]Department of Pathology, Brain Research Institute, University of Niigata, Niigata, 951-8585, Japan

*These two authors contributed equally to this study.

Correspondence and requests for materials should be addressed to M.F. (e-mail: fujiimas@med.niigata-u.ac.jp)

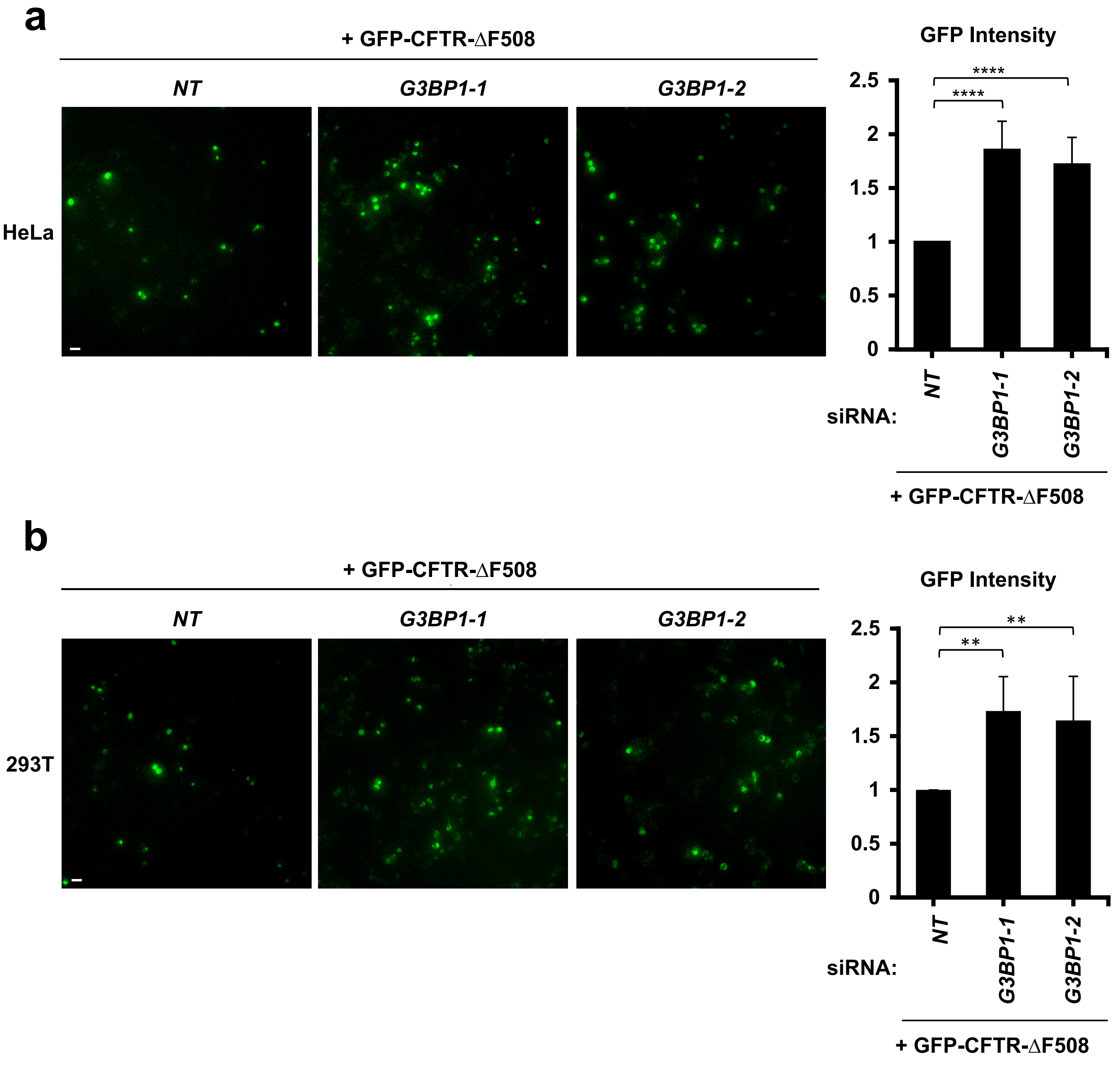

Supplementary Figure S1. G3BP1-knockdown increases the GFP-CFTR-ΔF508 aggregation. (**a, b**) HeLa (**a**) or 293T cells (**b**) were transfected with two different G3BP1-siRNAs (*G3BP1-1, G3BP1-2*) or a control (*NT*) and then transfected with the GFP-CFTR-ΔF508 plasmid. Live cell images were acquired using a fluorescent microscope. The bars indicate 20 μm. The ratio of the GFP intensity in the cells transfected with G3BP1-siRNA relative to those of the control cells was presented as the mean and standard deviation (SD). $**P < 0.01$; $****P < 0.0001$.

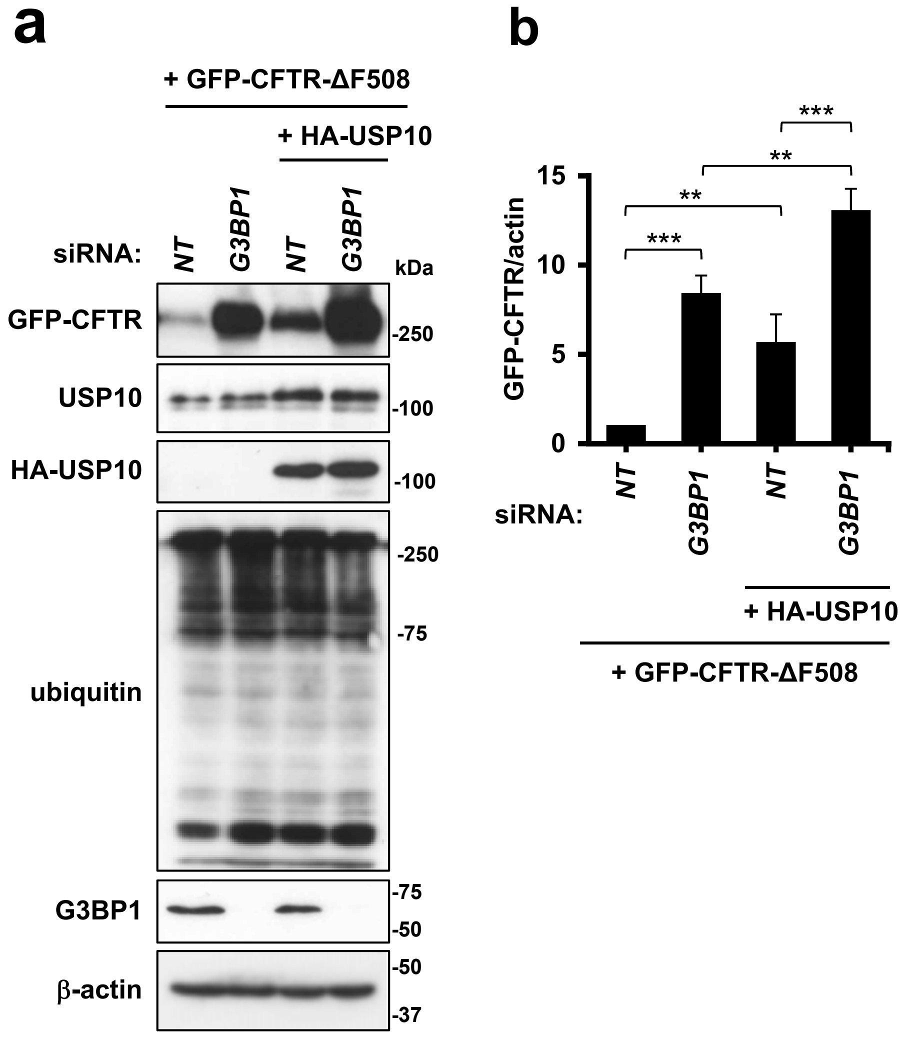

Supplementary Figure S2. USP10 augments the G3BP1-knockdown-induced increase in the GFP-CFTR-ΔF508 protein level. (**a**) HeLa cells were transfected with G3BP1-siRNA (*G3BP1*) or control (*NT*), and the cells were then transfected with GFP-CFTR-ΔF508 encoding plasmid together with HA-tagged USP10 or empty vector. Whole-cell lysates were subjected to a Western blot analysis using anti-GFP (CFTR), anti-USP10, anti-HA-tag, anti-ubiquitin, anti-G3BP1 and anti-β-actin antibodies. (**b**) The ratios of GFP-CFTR bands relative to β-actin were presented as the means and SD from three experiments. $^{**}P < 0.01$, $^{***}P < 0.001$.

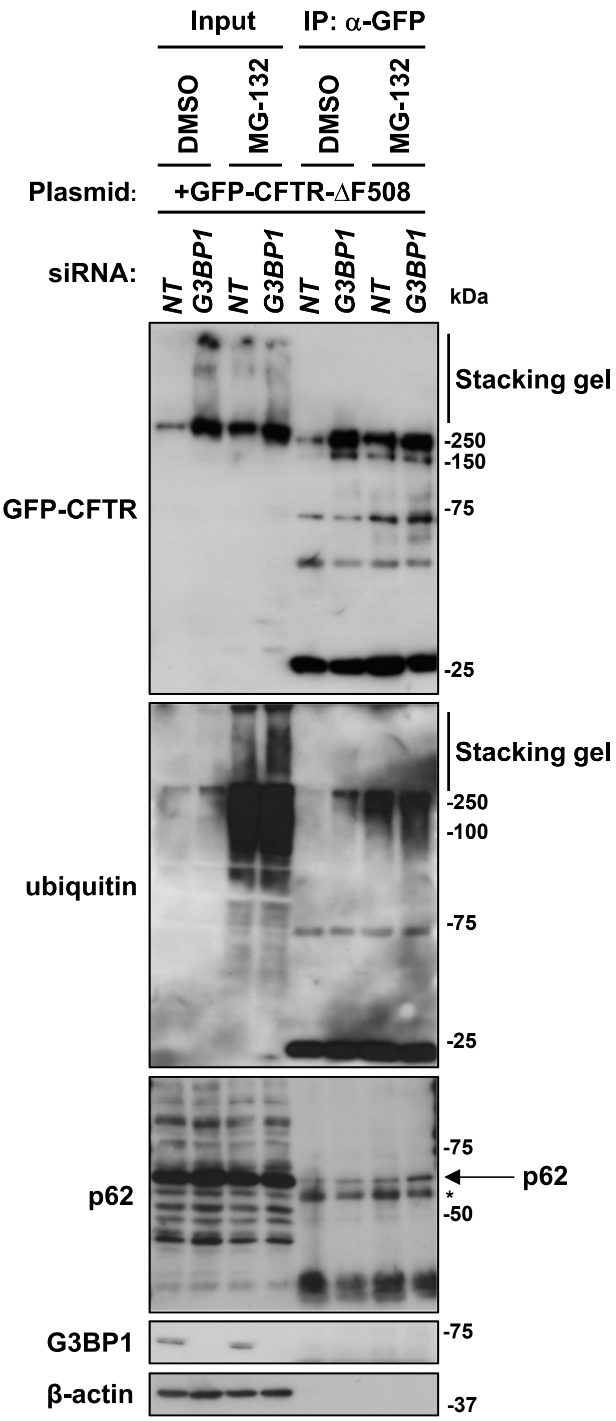

Supplementary Figure S3. G3BP1-knockdown increases ubiquitination of CFTR-ΔF508. HeLa cells were transfected with G3BP1-siRNA (*G3BP1*) or the control (*NT*) and transfected with the GFP-CFTR-ΔF508 plasmid. Cells were then treated with 5 μM MG-132 or DMSO for 6 h prior to harvesting. Cell lysates were immunoprecipitated with anti-GFP antibody or normal mouse IgG. The cell lysate (Input) and immunoprecipitate (IP) were subjected to a Western blot analysis with anti-GFP (CFTR), anti-ubiquitin, anti-p62, anti-G3BP1 and anti-β-actin antibodies. Asterisk indicates non-specific band.

Figure 1a

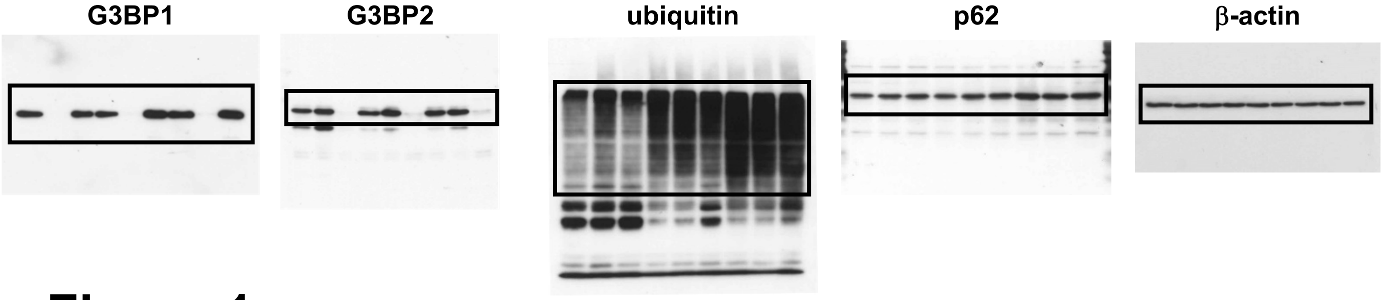

Figure 1c

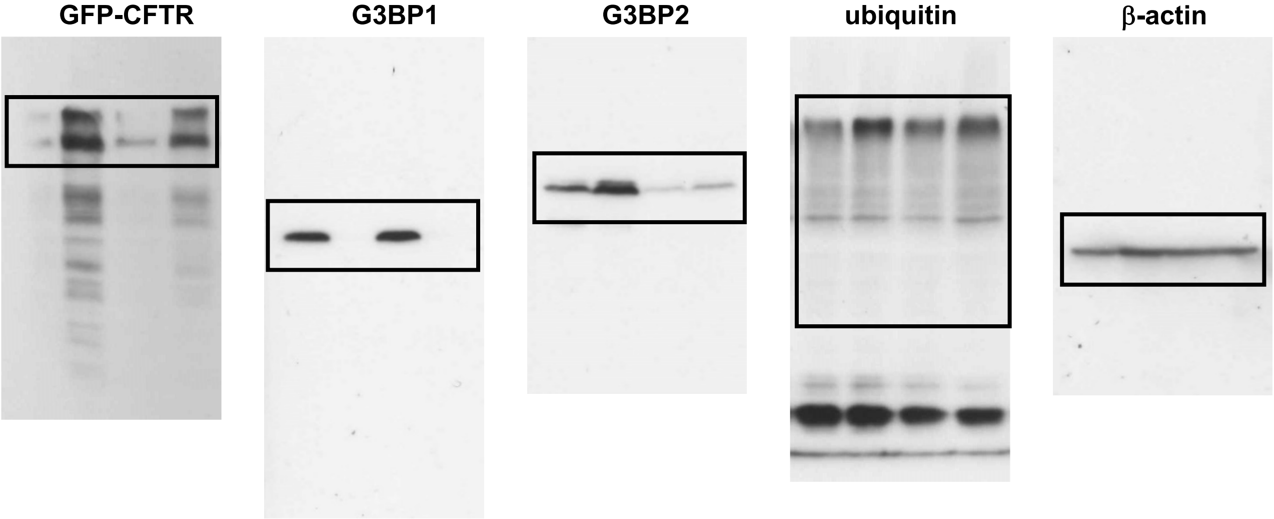

Figure 1f

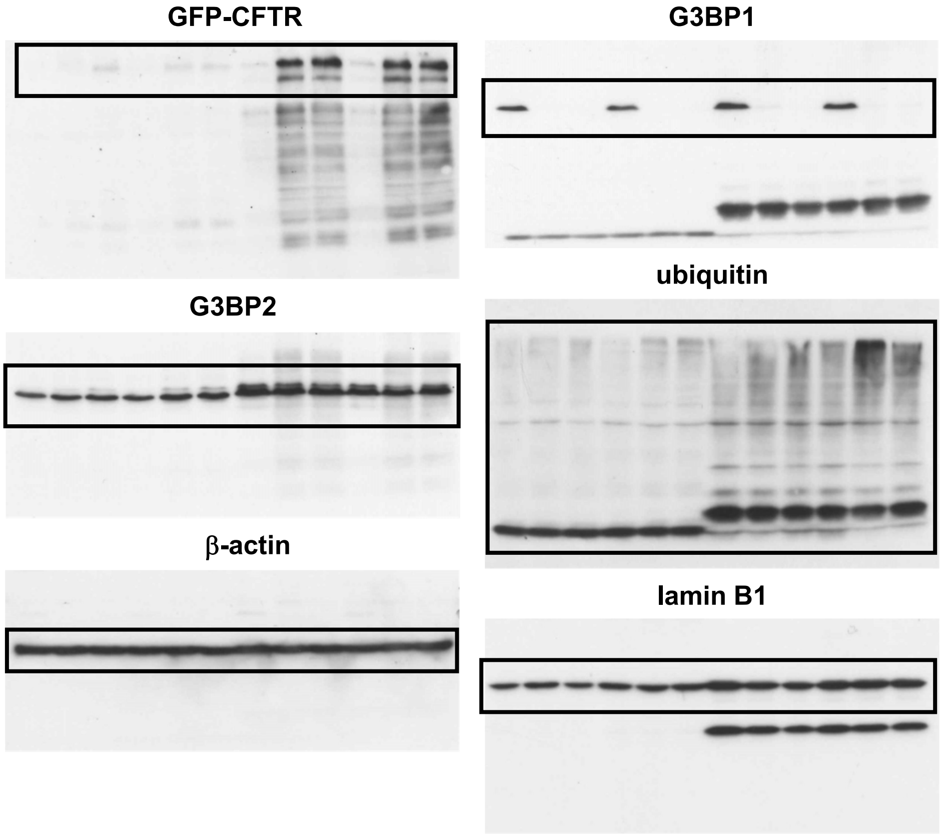

Supplementary Figure S4. Full length blots shown in Figure 1.

Figure 2a

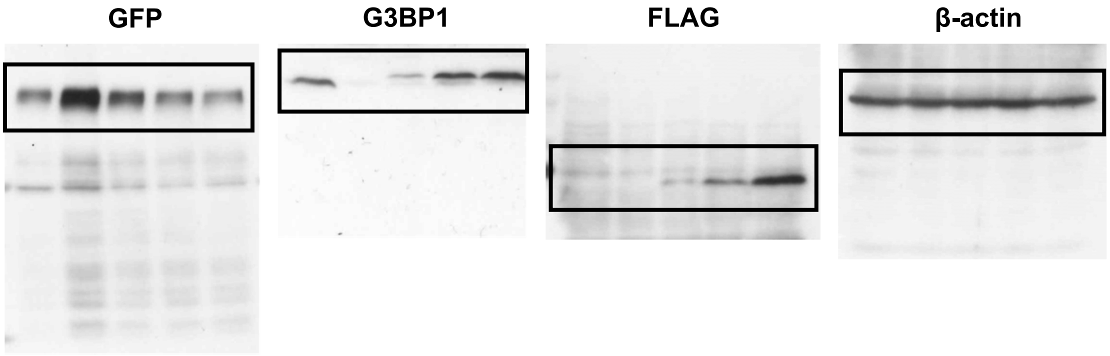

Figure 2c

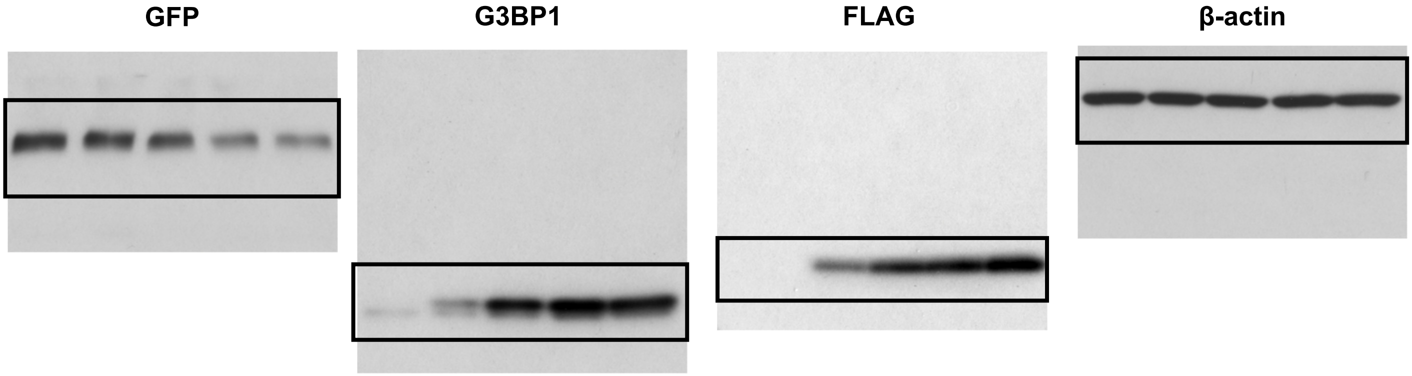

Supplementary Figure S5. Full length blots shown in Figure 2.

Figure 3a

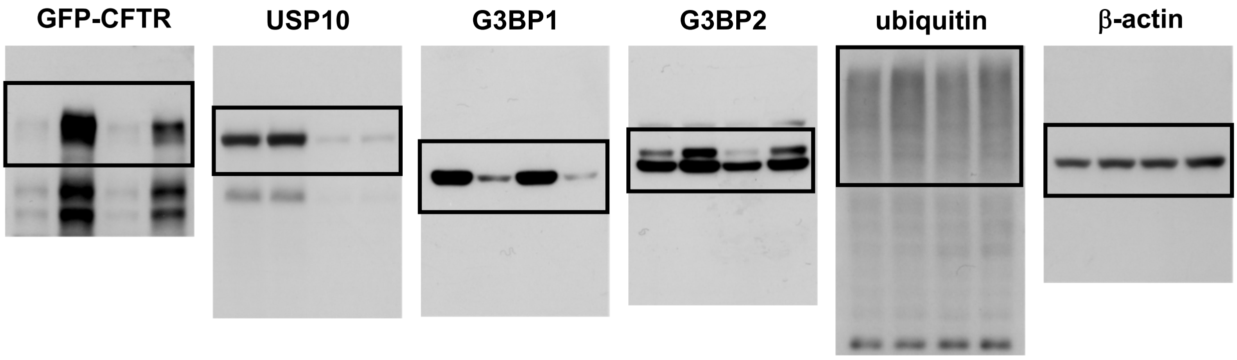

Figure 3d

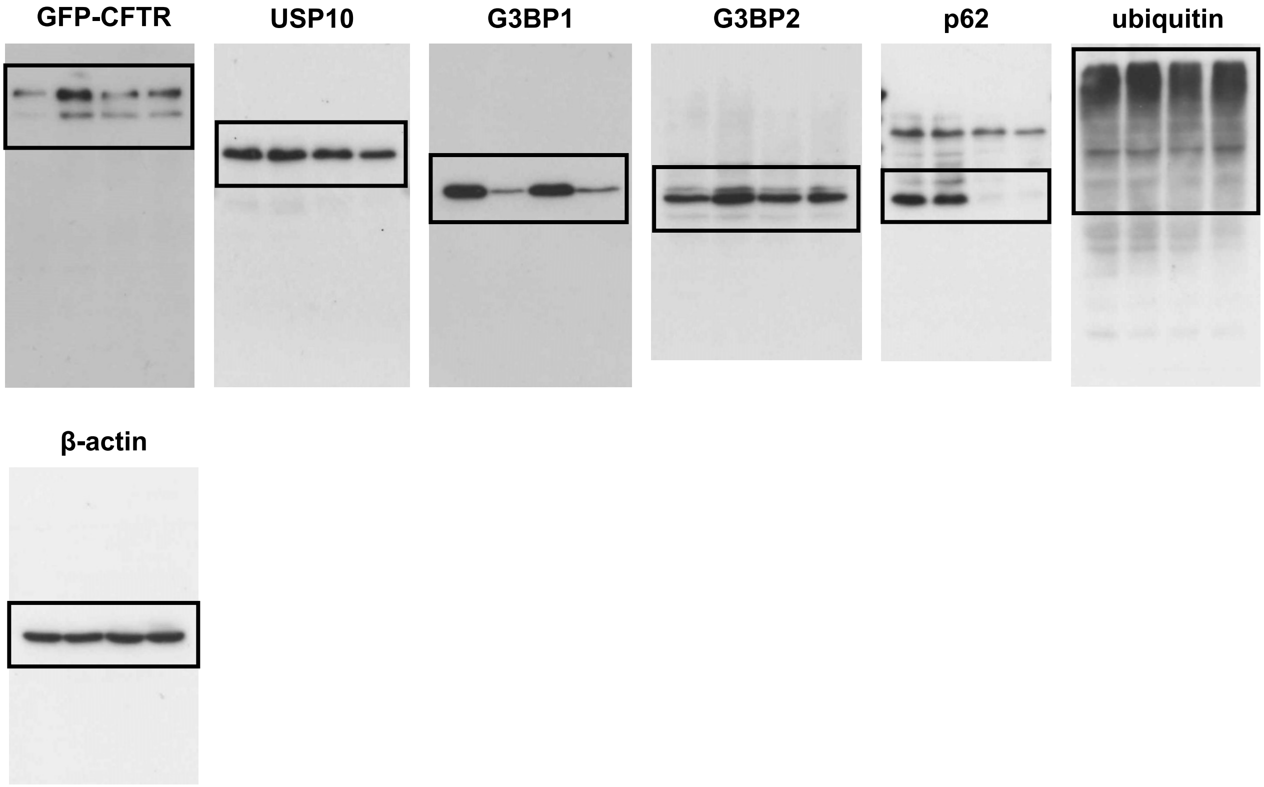

Supplementary Figure S6. Full length blots shown in Figure 3.

Figure 4a

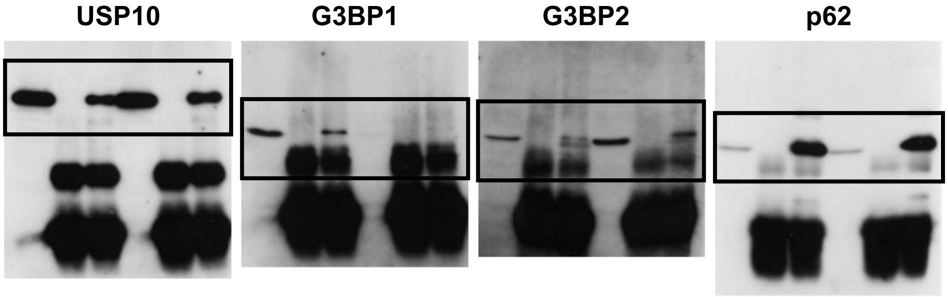

Figure 4b

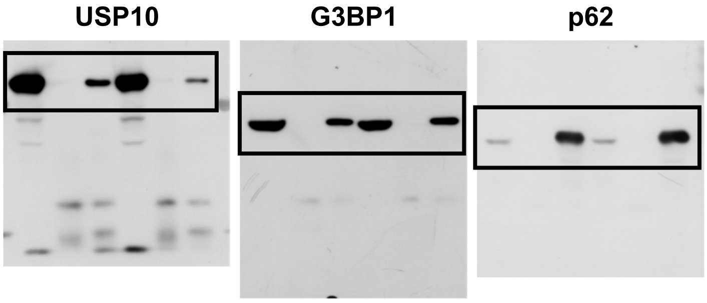

Figure 4c

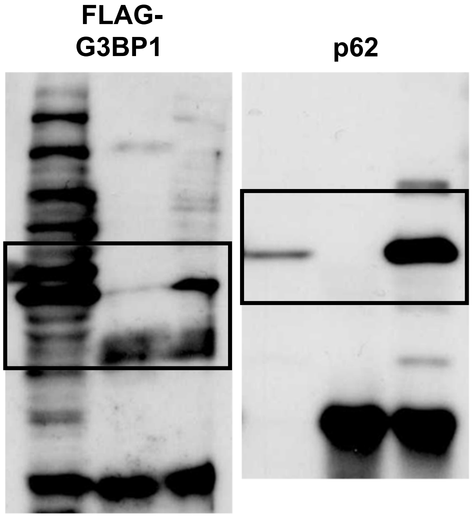

Figure 4d

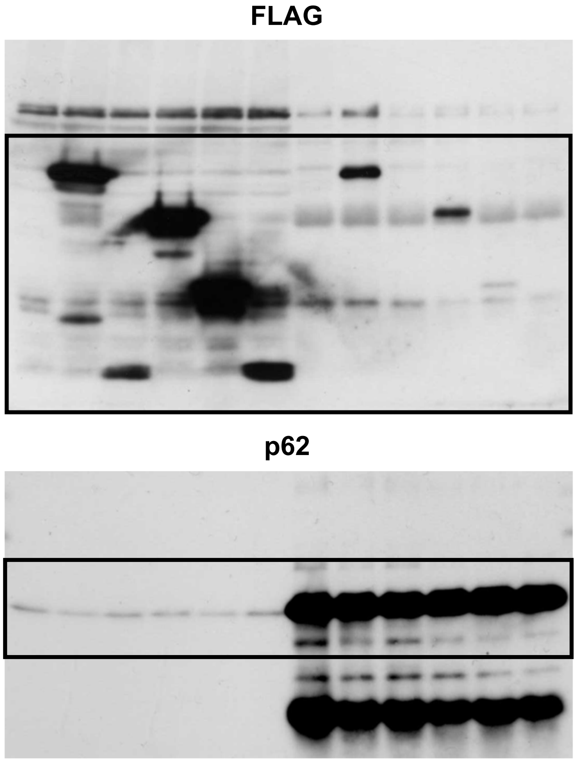

Supplementary Figure S7. Full length blots shown in Figure 4.

Figure 5a

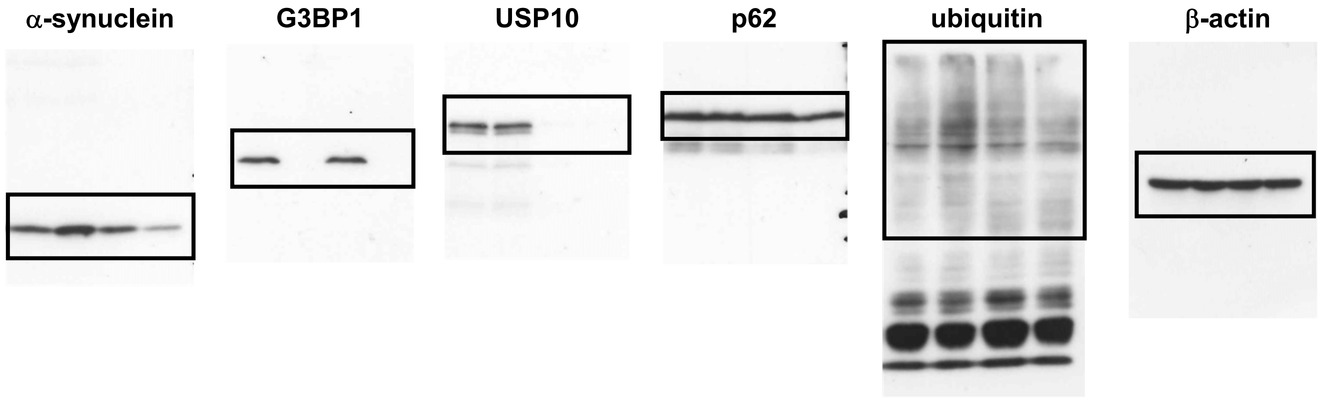

Figure 5c

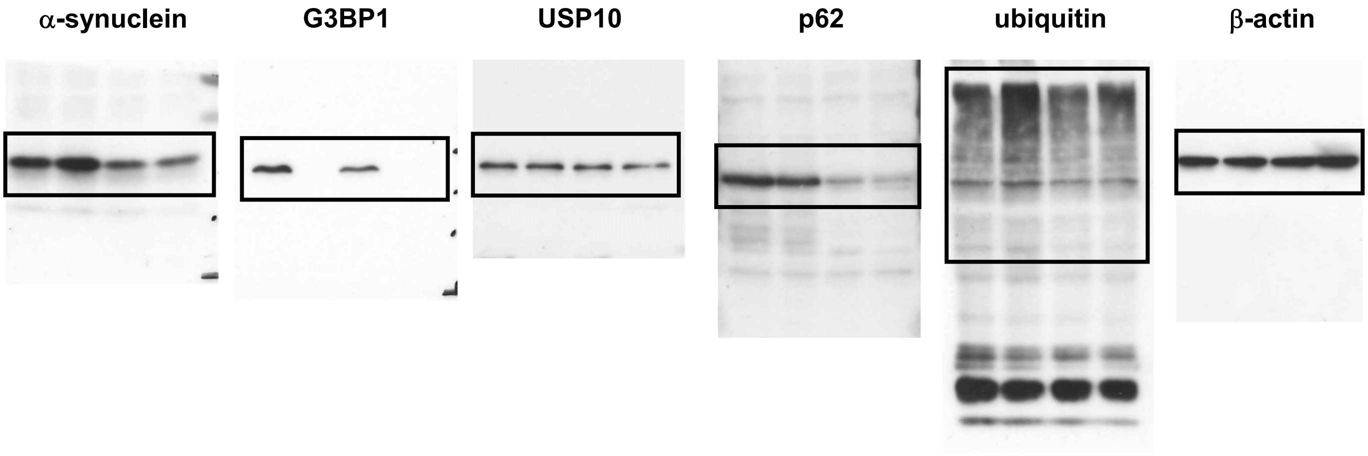

Supplementary Figure S8. Full length blots shown in Figure 5.

Figure 6a

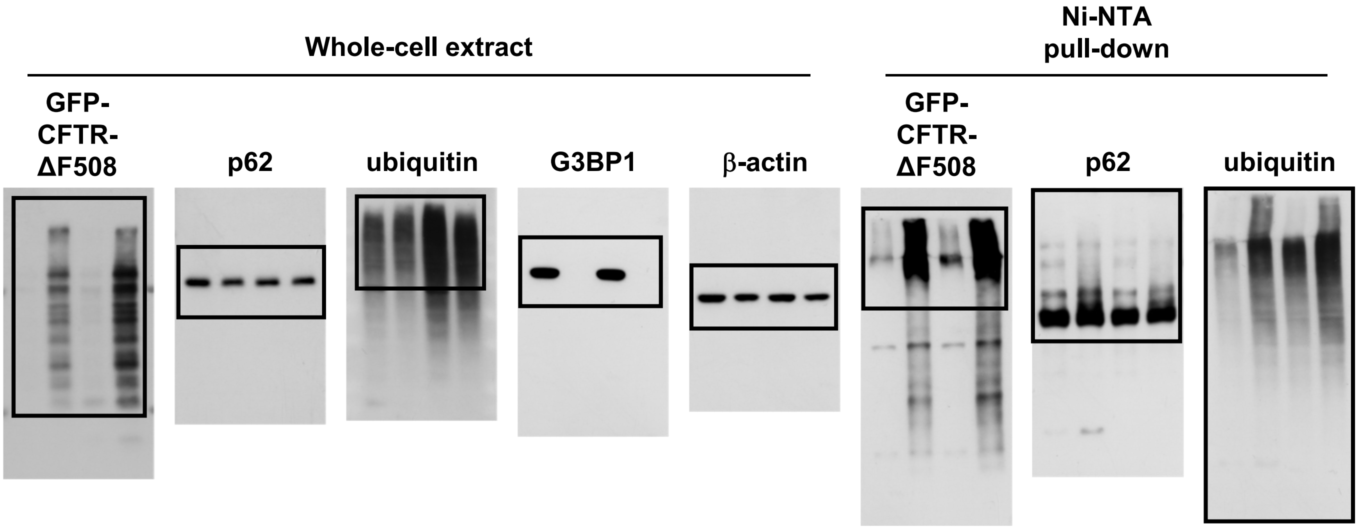

Figure 6b

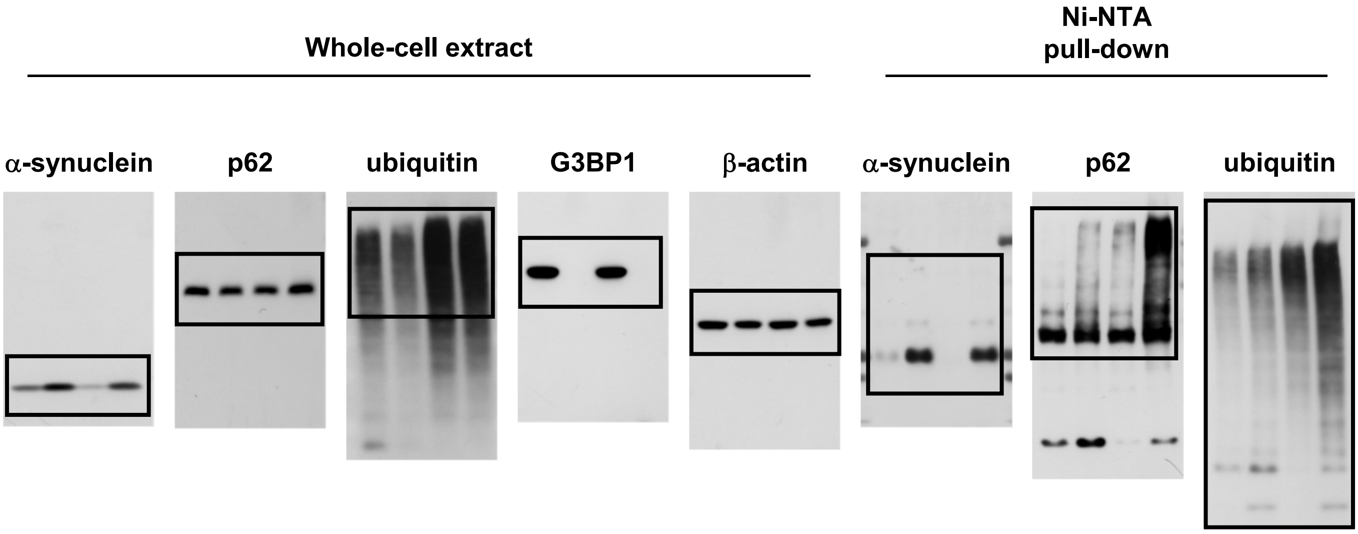

Supplementary Figure S9. Full length blots shown in Figure 6.

Figure 7a

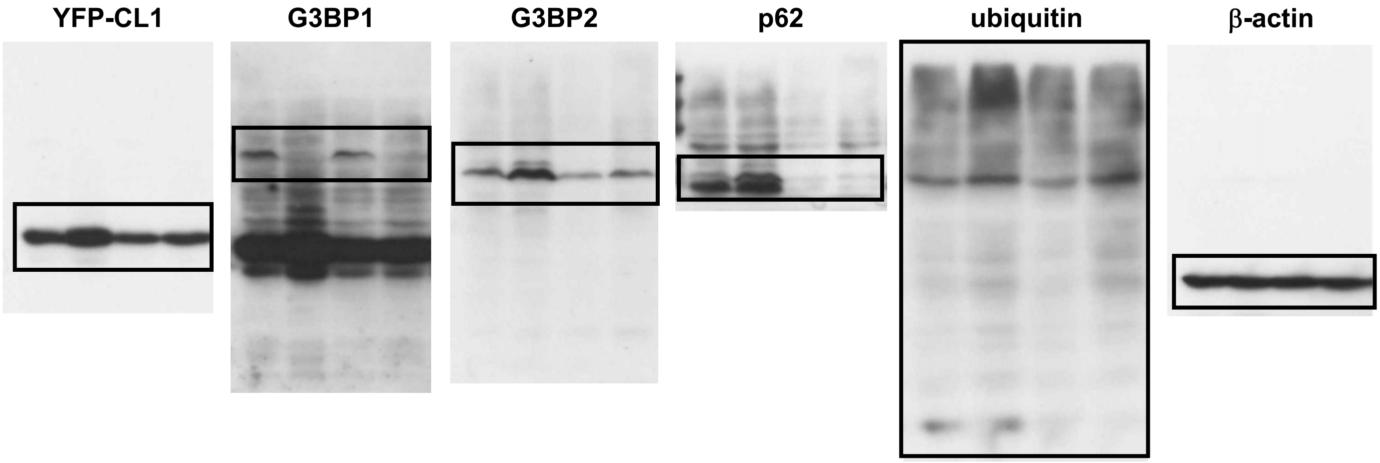

Supplementary Figure S10. Full length blots shown in Figure 7.

Figure 8a

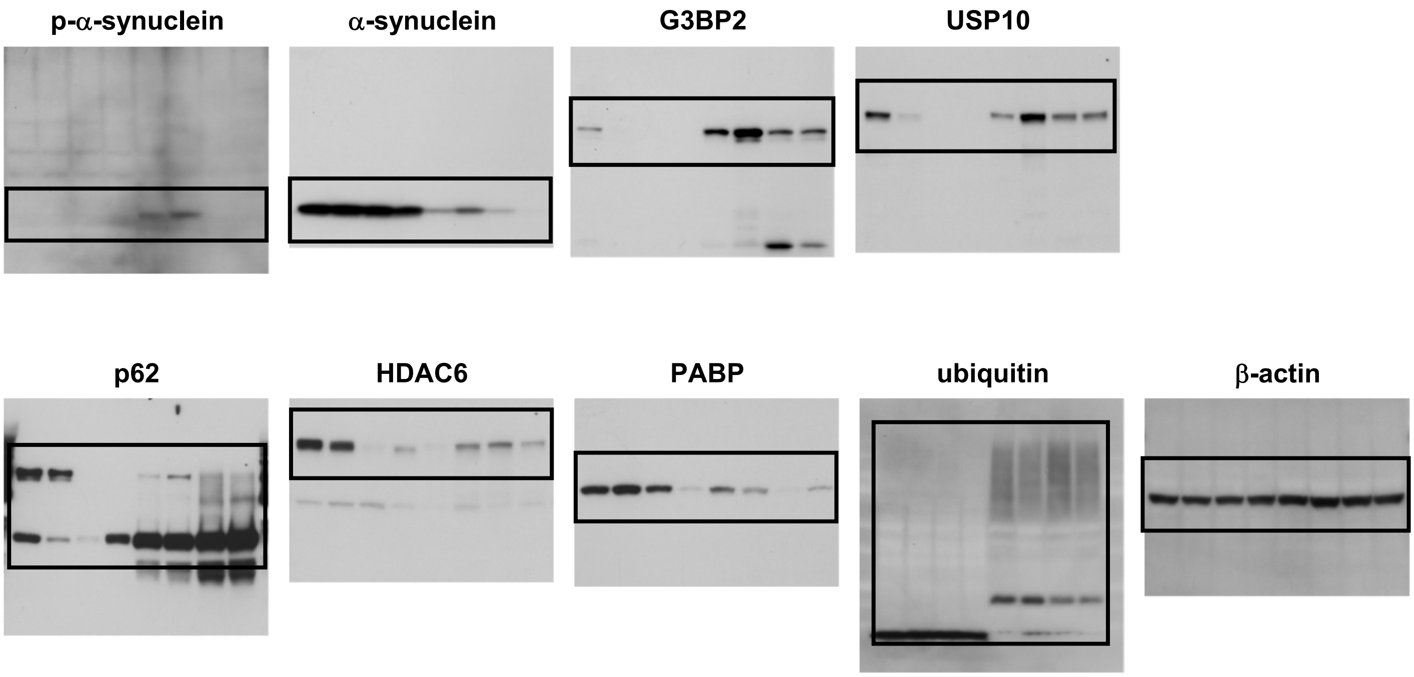

Figure 8b

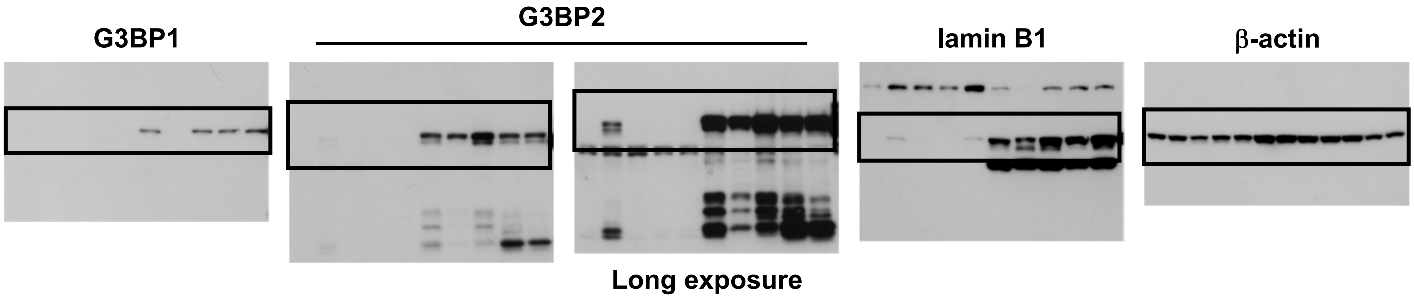

Supplementary Figure S11. Full length blots shown in Figure 8.

Supplementary Figure S2a

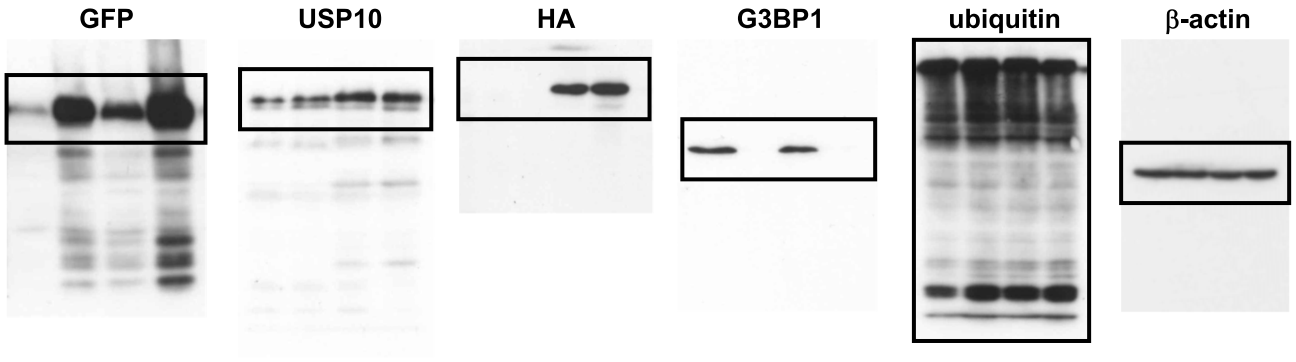

Supplementary Figure S12. Full length blots shown in Supplementary Figure S2.

Supplementary Figure S3

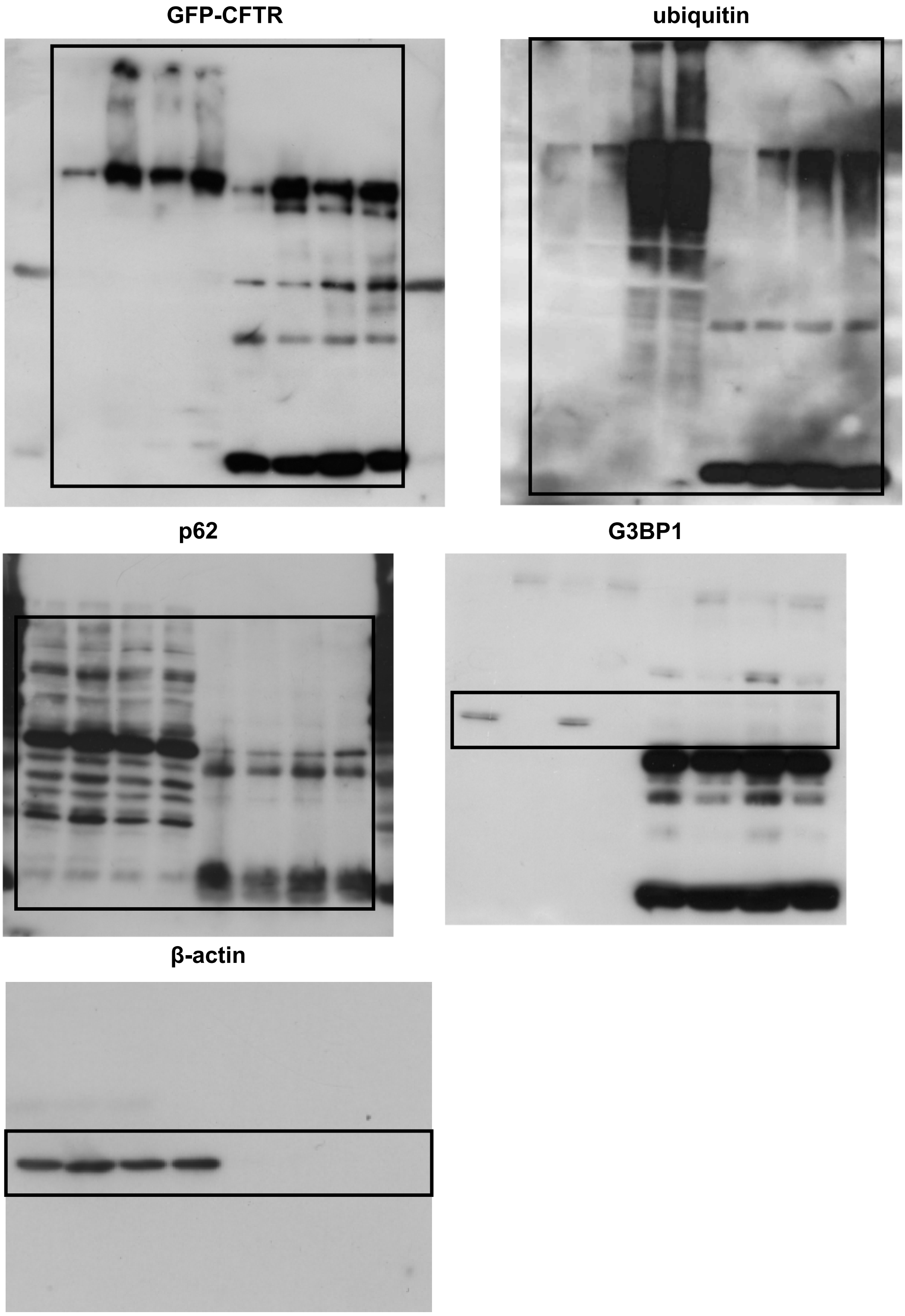

Supplementary Figure S13. Full length blots shown in Supplementary Figure S3.

Supplementary Method

Measurement of GFP intensity in living cells

To measure CFTR-aggregation-positive living cells, cells were transfected with G3BP1-siRNA and then the GFP-CFTR-ΔF508 plasmid described in the Methods. The GFP intensity in living cells was measured from more than 300 cells in 10 random fields from two different cell samples using a fluorescent microscope the BZ-II analyzer software program. The ratio of the GFP intensity in cells transfected with G3BP1-siRNA relative to those of the control cells was presented as the mean and standard deviation (SD).
